# Non-sutural basicranium-derived cells undergo a unique mineralization pathway via a cartilage intermediate *in vitro*

**DOI:** 10.7717/peerj.5757

**Published:** 2018-10-23

**Authors:** Holly E. Weiss-Bilka, Justin A. Brill, Matthew J. Ravosa

**Affiliations:** 1 Department of Biological Sciences, University of Notre Dame, Notre Dame, IN, United States of America; 2 Department of Aerospace and Mechanical Engineering, University of Notre Dame, Notre Dame, IN, United States of America; 3 Department of Anthropology, University of Notre Dame, Notre Dame, IN, United States of America

**Keywords:** Basicranium, Osteoblast, Mineralization potential, Endochondral, Embryology, Skull, Mouse, Mammals, BMP6

## Abstract

The basicranium serves as a key interface in the mammalian skull, interacting with the calvarium, facial skeleton and vertebral column. Despite its critical function, little is known about basicranial bone formation, particularly on a cellular level. The goal of this study was therefore to cultivate a better understanding of basicranial development by isolating and characterizing the osteogenic potential of cells from the neonatal murine cranial base. Osteoblast-like basicranial cells were isolated, seeded in multicellular aggregates (designated micromasses), and cultured in osteogenic medium in the presence or absence of bone morphogenetic protein-6 (BMP6). A minimal osteogenic response was observed in control osteogenic medium, while BMP6 treatment induced a chondrogenic response followed by up-regulation of osteogenic markers and extensive mineralization. This response appears to be distinct from prior analyses of the calvarium *and* long bones, as basicranial cells did not mineralize under standard osteogenic conditions, but rather required BMP6 to stimulate mineralization, which occurred via an endochondral-like process. These findings suggest that this site may be unique compared to other cranial elements as well as the limb skeleton, and we propose that the distinct characteristics of these cells may be a function of the distinct properties of the basicranium: endochondral ossification, dual embryology, and complex loading environment.

## Introduction

Despite a large degree of variation in shape and characteristics, mammalian bone has typically been viewed as a homologous material possessing equivalent properties across all skeletal regions (Eames & Helms, 2004). This interpretation, while appealing in its simplicity, fails to account for the many complexities of the skeleton, particularly within the skull. Cranial bones vary with respect to embryological origin (paraxial mesoderm, neural crest) (Noden, 1983; Chai et al., 2000; Jiang et al., 2002; Szabo-Rogers et al., 2010; Som & Naidich, 2013) and ossification mode (intramembranous, endochondral) (Morriss-Kay & Wilkie, 2005; Richtsmeier & Flaherty, 2013) and are subjected to vastly different patterns and magnitudes of mechanical loads (constant vs. cyclic; low vs. high magnitude) *in vivo* (Herring & Teng, 2000; Ravosa, Johnson & Hylander, 2000; Ravosa et al., 2010; Fong et al., 2003; Ross & Metzger, 2004). Given these differences, it follows that cranial bones are likely to have distinct properties depending on their anatomical location.

It has long been recognized that osteoblasts from the skull (parietal) respond disparately to applied loading when compared to osteoblasts from the limb skeleton (ulna) of rats (Rawlinson et al., 1995), indicating that calvarial bone is distinct from the limb skeleton. Furthermore, recent studies have revealed differences in the mineralization potential and regenerative abilities of osteoblasts derived from neural crest-derived frontal bones compared to paraxial mesoderm-derived parietal bones (Quarto et al., 2010; Senarath-Yapa et al., 2013), suggesting that additional variation exists within a given cranial region. The concept of regional and within-region variation among cranial elements in mammals is even more compelling given recent evidence of multi-scale architectural heterogeneity in the rabbit skull (Franks et al., 2016; Franks et al., 2017). Taken together, these data suggest that the skull is much more complex than previously recognized and that the properties of individual cranial bones, and the cells comprising them, may be site-specific.

The basicranium, or cranial base, is unique among other skull elements such as the calvarium and much of the facial skeleton in that it arises via the process of endochondral ossification (Richtsmeier & Flaherty, 2013). In this process, mesenchymal stem cells condense and form a cartilage intermediate prior to mineralization and bone formation, rather than direct ossification of the cellular condensation through the intramembranous pathway (Eames, De La Fuente & Helms, 2003). The cranial base also has a dual embryological origin, arising from cells of both neural crest, anterior to the pituitary gland (basisphenoid), and paraxial mesoderm, posterior to the pituitary gland (basioccipital) (Szabo-Rogers et al., 2010; Som & Naidich, 2013). Structurally, the basicranium serves as a central interface in the skull, interacting with several cranial structures including the calvarium, inner ear, facial skeleton and vertebral column, and providing a conduit for cranial nerves to connect with the rest of the body (Ross & Ravosa, 1993; Lieberman, Ross & Ravosa, 2000; Richtsmeier & Flaherty, 2013). It forms the floor of the neurocranium, supporting and protecting the brain while also providing sufficient room for its growth (Lieberman, Ross & Ravosa, 2000; Richtsmeier & Flaherty, 2013). Thus, the basicranium has come to be known as the “keystone of the skull,” (Jeffery, 2003) and is thought to play a critical role in the coordinated development of the braincase and facial skeleton (Nie, 2005; López et al., 2008; Richtsmeier & Flaherty, 2013). Given the extent of its role in skull ontogeny, surprisingly little is known about the osteogenic mechanisms driving cranial base formation, particularly on a cellular level.

In an effort to better understand early postnatal bone formation in the cranial base, and to further establish the presence of heterogeneity among cranial sites, the objectives of this study were to isolate basicranial osteoblast-like cells (bOBs) from neonatal mice and characterize the behavior of bOBs *in vitro* by assessing their proliferative capacity and mineralization potential. Due to the unique characteristics of the cranial base (dual embryological origin of cells; endochondral ossification), we hypothesized that bOBs would exhibit disparate behaviors when compared to prior research on the well-characterized OBs of the calvarium.

Bone morphogenetic protein 6 (BMP6) was selected as a potential agent for evaluating variation in cranial mineralization because it is expressed in the developing basioccipital bone (Iwasaki, Le & Helms, 1997) as well as in pre-hypertrophic and hypertrophic chondrocytes in the growth plate of long bones (Vortkamp et al., 1996; Eames, De La Fuente & Helms, 2003). BMP6 transcripts have also been detected in hypertrophic chondrocytes in the early postnatal murine cranial base (Kettunen et al., 2006). BMP6 binds the type II BMP receptor (BMPR2), which then associates with a type I BMP receptor and triggers the BMP signaling pathway. Downstream targets of the BMP/BMP receptors are critical to the endochondral pathway (Retting et al., 2009; Yang et al., 2010), underscoring the utility of BMP6 in basicranial development. Moreover, *in vitro* studies have demonstrated that exogenous BMP6 stimulates chondrocyte maturation in cells isolated from chicken embryo sterna (Grimsrud et al., 1999) and induces rapid maturation and mineralization in chick limb-bud micromass cultures (Boskey et al., 2002), further implicating it as a mediator of the endochondral pathway. It has also been shown to induce both chondrogenesis and osteogenesis in adult adipose-derived stem cells cultured in 3D pellet or 2D monolayer, respectively (Kemmis et al., 2010).

## Experimental Procedures

### Animal model

To investigate early postnatal bone formation in the cranial base, mice were chosen as a model organism because they share many common features with humans and other mammals. Importantly, bones of the braincase share the same embryology and arise by the same ossification pathways in humans and mice (Rossant & Tam, 2002). Additionally, the murine cranial base develops in a sequence similar to that of humans and rats, as its growth and ossification progress in a caudal to rostral sequence (Nie, 2005; Kettunen et al., 2006). Hence, while the remodeling process differs between the two species, the pattern and characteristics of cranial bone development are remarkably similar.

### Cell isolation

**Figure 1:**
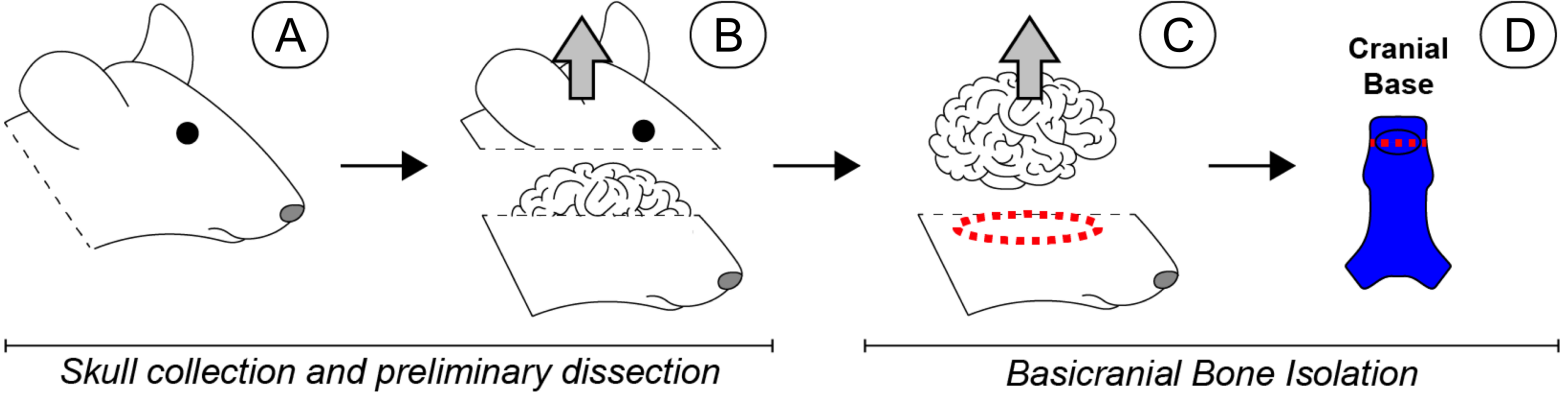
Scope-mediated dissection and isolation of basicranial bone. (A) Four day-old mouse pups were euthanized and the heads collected and rinsed in PBS. (B) The calvarium was cut away and removed. (C) The brain was then retracted, and the cranial base dissected away from the remaining skull with microdissection scissors (red dashed region). (D) Soft tissues and sutures were carefully removed, and the basioccipital and basisphenoid were separated to remove the spheno-occipital synchondrosis (red dashed line), leaving non-sutural cranial bone (bright blue). Special care was taken to remove the pituitary gland, which was approximately located in the region indicated by the black oval. Once dissected, all basicranial bone fragments were pooled for subsequent cell isolation. Please note that a human-like brain is depicted for heuristic purposes.

For each experiment, non-sutural primary osteoblast-like cells (bOBs) were isolated from the basicranium of 20–22 postnatal day 4 (P4) ICR mouse pups (Fig. 1). All such work was approved by the University of Notre Dame IACUC (protocol #17-10-4138 to MJR). First, the calvarium was cut away and the brain was removed with an inoculating loop. The basisphenoid and basioccipital regions of the cranial base were then separately dissected from the remaining skull using microdissection scissors, soft tissues were carefully removed, and sutures and synchondroses cut away. Cleaned bones were rinsed in sterile PBS (Corning Inc., Corning, NY, USA) and then minced into smaller pieces for further processing. Partial digestion was achieved via a series of incubations in type II collagenase (Worthington Biochemical Corporation, Lakewood, NJ, USA), 0.25% trypsin (Thermo Fisher Scientific, Life Technologies, Waltham, MA, USA) and EDTA (Thermo Fisher, Fisher Scientific) as described in Bakker & Klein-Nulend (2012).

In order to maximize cell yield, and therefore the potential analyses that could be performed for a given experiment, we isolated osteoblasts using two standard methods: (1) cells released from basicranial bone fragments during collagenase digests were collected via centrifugation (Bakker & Klein-Nulend, 2012), and (2) remaining resident cells were encouraged to migrate out of the partially digested bone fragments (termed ‘outgrowth’) by placing all remaining pieces directly on tissue culture plastic and cultured in growth medium (Ecarot-Charrier et al., 1983; Ecarot-Charrier et al., 1988). Basicranial bone fragments processed via the first method were subjected to four rounds of collagenase digestion, 20 min per digest, with periodic agitation. Cells from digestions 3 and 4 were collected via centrifugation and plated into 6-well Primaria dishes for expansion (Falcon Brand, Corning, Corning, NY). The remaining bone fragments processed via the second method were rinsed with DMEM (Life Technologies) prior to placement in separate wells of 6-well Primaria dishes for outgrowth. In terms of subsequent experiments, cells obtained from rounds 3 and 4 of collagenase digestion (populations 3 and 4) were pooled with the cells obtained from bone fragment outgrowth (population 5) at the first passage to form a single osteoblast-like cell population.

### Expansion

Cells were cultured in growth medium (GM) comprised of DMEM-LG (Thermo Fisher, Life Technologies) with 10% FBS (Atlas Biologicals, Fort Collins, CO, USA), 1% Penicillin-Streptomycin (Pen-Strep; Thermo Fisher, Life Technologies) and 100 µg/mL ascorbic acid (Sigma-Aldrich, St. Louis, MO, USA). Due to the small size of the basisphenoid and basioccipital of P4 mice, and therefore a relatively small cell yield, we did not distinguish between basioccipital and basisphenoid cells, but rather pooled them into a single bOB cell population. Medium was changed every other day. Cells were passaged when they reached near-confluence and were re-plated at 2,250–2,500 cells/cm^2^. Because cells were cultured in the continuous presence of ascorbic acid, a collagenase pre-treatment (Gallagher, 2003) was applied prior to passaging. Cells were washed twice with serum-free DMEM, and were then treated at 37 °C with DMEM containing 25 U/mL type II collagenase and 2 mM CaCl_2_ (Sigma) for 2 h, agitating every 30 min. Treated cells were washed twice with PBS and detached via incubation in 0.25% trypsin-EDTA at 37 °C (Thermo Fisher, Life Technologies). At the first passage, cells from digests 3, 4 and 5 were pooled. Cells from passage 2 were used for all experiments.

### Plating and growth of MMs

Basicranial OBs from passage 2 were collected via centrifugation, counted, and assessed for viability with Trypan Blue (Sigma). For 3D micromass (MM) culture, multi-layered aggregates of bOBs were formed in 24-well plates by seeding 100,000 cells in 15 µL DMEM-LG supplemented with 10% FBS and 1% Pen-Strep. Once cells had begun to condense, 0.5 mL fresh GM was added to each well. Treatment media were applied the following day. The micromass culture system was chosen over standard monolayer culture because it allows for a multilayer structure that better mimics cell–cell attachments and 3D morphologies present *in vivo*, which is important in modeling early skeletogenesis (Klumpers, Mooney & Smit, 2015). Furthermore, micromass culture has been shown to enhance the differentiation of primary osteoblast-like cells compared to standard monolayer culture (Gerber et al., 2005).

Preliminary studies demonstrated that neither standard osteogenic medium containing ascorbic acid and *β*-glycerophosphate nor standard chondrogenic medium containing TGF *β* was able to induce appreciable tissue formation in basicranial control OB MMs within 6 weeks of treatment. Thus, two induction media were selected for this study: osteogenic medium served as a control (OM: GM supplemented with 10 mM β-glycerophosphate (EMD Millipore, Billerica, MA, USA)), and BMP6 medium was the treatment condition (BMP6: OM further supplemented with 100 ng/mL bone morphogenetic protein-6 (PeproTech, Rocky Hill, NJ, USA)). The BMP6 formulation chosen herein is based on a prior study of murine adipose-derived stem cells that produced an osteogenic response in monolayer and a chondrogenic response in pellet culture (Kemmis et al., 2010). MMs were cultured in induction media for up to 6 weeks with three media changes per week.

### Biochemical and molecular analysis

Cellular activity and matrix deposition was assessed at the time of plating (week 0) and following 2, 4 and/or 6 weeks of induction in treatment media. To gauge proliferation, total DNA content was quantified at 0, 2, 4 and 6 weeks with Quant-iT™ PicoGreen^®^ dsDNA Assay Kit (Thermo Fisher), per manufacturer’s instructions (*n* = 3 replicates).

Chondrogenic matrix was evaluated by selectively staining sulfated glycosaminoglycans (sGAGs) in 4 and 6-week MMs with 1% Alcian Blue solution at pH 1.0. MMs were washed twice with PBS, fixed in 100% ethanol for 20 min, and then stained with Alcian Blue for 30 min at room temperature. MMs were washed with DI H_2_O 3 times prior to imaging and quantification. Bound dye was extracted for quantification by applying 6 M guanidine hydrochloride (Sigma) to MMs overnight at room temperature. The optical density of re-suspended dye was measured at 600 nm in a clear 96-well plate (two replicate experiments) (Woods, Wang & Beier, 2005). To confirm trends observed by Alcian Blue staining, an additional experiment was performed to measure sGAG content in MM lysates at 6 weeks and in conditioned culture medium samples at weekly time points from week 1 through week 6 with the Blyscan Glycosaminoglycan Standard Assay Kit (Accurate Chemical & Scientific Corporation, Westbury, NY, USA). Lysate values were reported as total sGAG as well as sGAG normalized to the DNA content of corresponding sGAG samples (*n* = 3 replicates) to assess the average sGAG per cell.

Alkaline Phosphatase (ALP) was used as an early osteogenic marker. Total ALP in MM lysates and conditioned culture media (two replicate experiments) was quantified with SensoLyte^®^ pNPP Alkaline Phosphatase Colorimetric Assay Kit (AnaSpec, Inc., Fremont, CA), as directed (*n* = 3). In addition, ALP content of lysates was normalized to the DNA content of corresponding ALP samples (*n* = 3) to provide a representative value for ALP content per cell.

To evaluate matrix mineralization, a late osteogenic marker, the calcium content of MM lysates was quantified with the Calcium Assay Kit (BioAssay Systems, Hayward, CA, USA), following manufacturer directions. Alternatively, calcified matrix was visualized by staining with 0.2% Alizarin Red S as previously described (Malladi et al., 2006). Briefly, cells were washed twice with PBS and once with deionized H_2_O prior to fixation in 100% ethanol. Alizarin Red S solution was then applied for 60 min at room temperature, after which cells were washed several times with deionized H_2_O.

Total RNA was isolated from MMs at weeks 0, 4 and 6 (*n* = 3) with RNeasy Micro Plus Kit (QIAGEN, Valencia, CA, USA), as directed. Chondrogenic markers (*Sox9, Col2a1*), proliferative marker (*PCNA*), hypertrophic marker (*Col10a1*), osteogenic markers (*ALP*, *Runx2*, *Col1a1*) and BMP signaling pathway marker (*BMPR2*) were evaluated. Chondrogenic genes were chosen because they complemented other chondrogenic analyses noted above. For instance, Sox9 is present in all chondrocytes except hypertrophic chondrocytes, and plays a critical role in endochondral bone formation in the skull (Mori-Akiyama et al., 2003). Examination of additional genes was not possible given the limited RNA from the micromasses. Complementary DNA was obtained by reverse transcription of 200 ng of total RNA using the High Capacity cDNA Reverse Transcription Kit. Quantitative PCR was performed on a Step One Plus System using Fast SYBR Green Master Mix (all from Thermo Fisher, Applied Biosystems), per manufacturer’s recommendations. Relative gene expression was calculated using the 2^−Δ*CT*^ method, using GAPDH as the housekeeping gene, or the 2^−ΔΔ*CT*^ method with week 0 as a reference condition.

### Data and statistics

Three replicate experiments were performed to assess repeatability of cell behavior. Excepting GAG and calcium assays, each assay was performed at least twice due to limited cell numbers. The GAG assay on cell lysates was performed once to confirm Alcian Blue quantitative results, and the calcium assay on cell lysates was performed once to quantify the trends observed via Alizarin Red staining. Data are represented as mean ± SD unless otherwise noted. Where applicable, statistical significance was assessed by *t*-test or by two-way ANOVA and Sidak’s post-test using GraphPad Prism Software (La Jolla, CA, USA). Significance was assessed as *p* < 0.05, and is indicated on graphs as follows: *p < 0.05, **p < 0.01, ***p < 0.001 and ****p < 0.0001.

## Results

### Primary culture of basicranial osteoblasts

**Figure 2:**
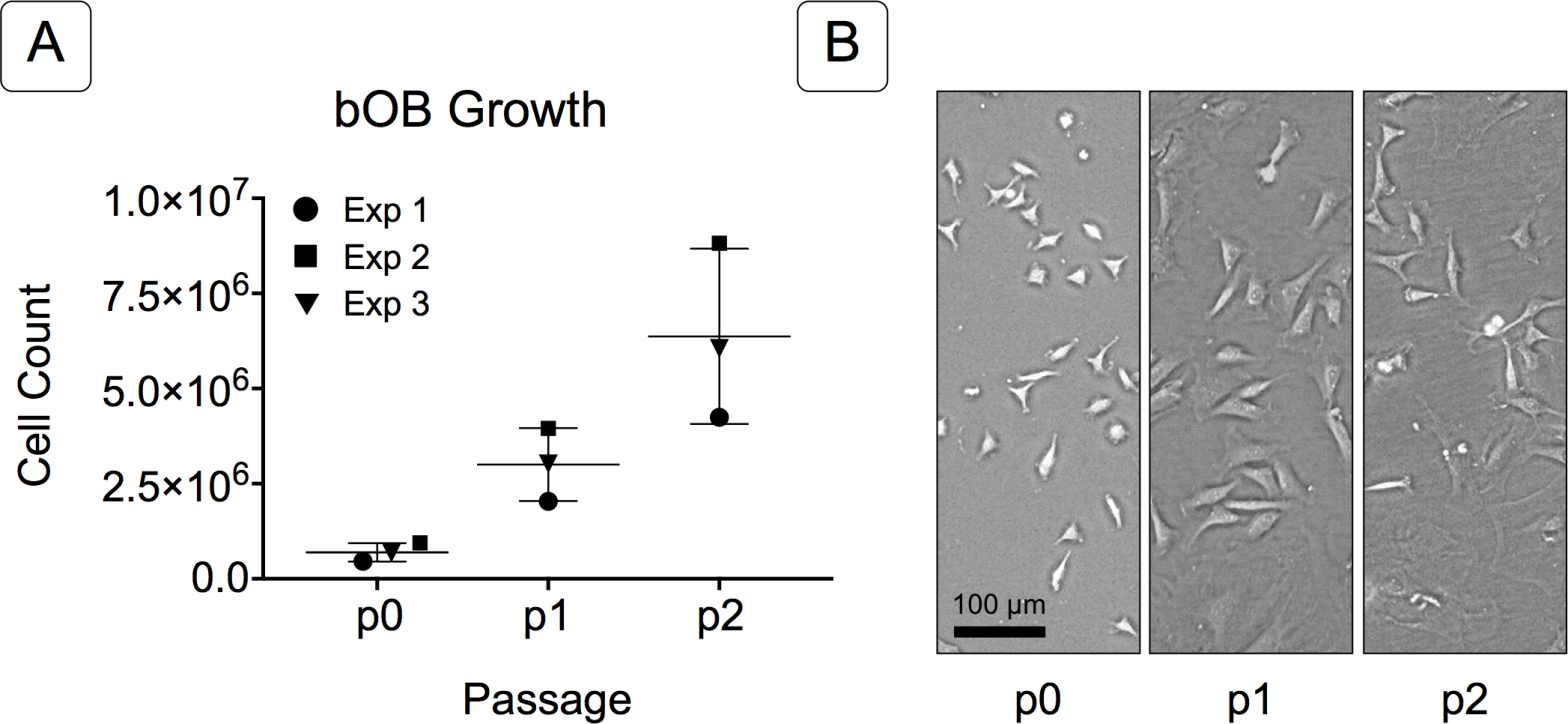
Growth of isolated basicranial osteoblasts (bOBs). (A) Cell counts at passages 0 through 2 (p0, p1, p2) of cell expansion for three replicate experiments. Matching symbols (square, inverted triangle, circle) denote bOB cell counts for each individual experiment; lines represent the mean cell count ± standard deviation for all 3 experiments. (B) Representative bright field images of isolated bOBs at each passage. Scale: 100 µm for all images. Cells from passage 2 were used for all induction studies.

**Figure 3:**
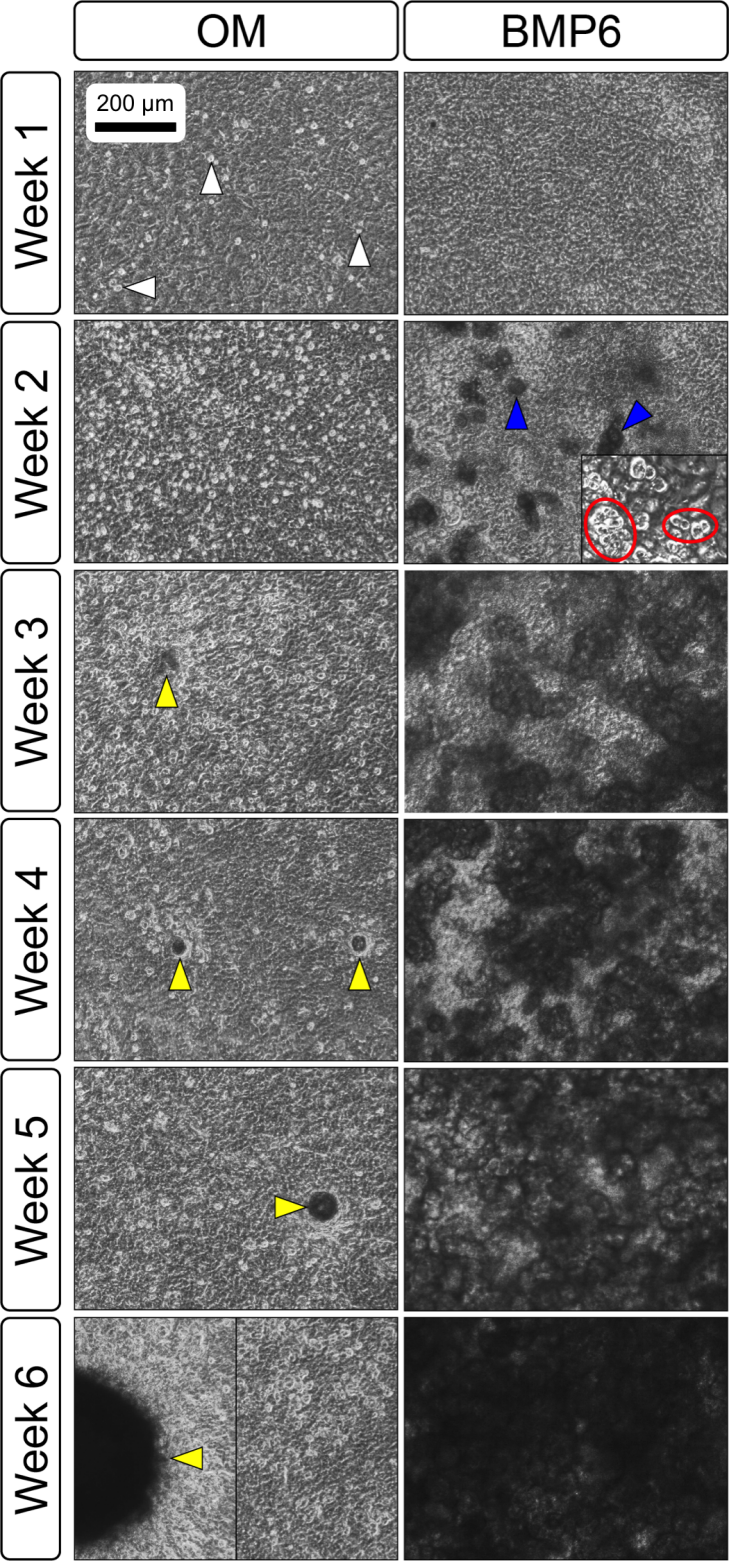
Osteogenic induction of micromasses (MMs). Bright field images of the central region of MMs treated with osteogenic (OM) or BMP6 supplemented (BMP6) induction medium over the course of 6 weeks. OM: Small, rounded cells began to appear as early as week 1 of induction (white arrowheads) and persisted through 6 weeks. Matrix accumulation was most evident in the form of nodules (yellow arrowheads), beginning at week 3. BMP6: Matrix deposition was evident as early as week 2 of induction, particularly in the pericellular region of enlarged cells displaying a hypertrophic morphology (blue arrowheads). Cells with a hypertrophic phenotype were also evident at the periphery of MMs (red ovals, week 2 inset). Extracellular matrix density increased with time in culture through week 6. Scale: 200 µm for all images.

Primary non-sutural osteoblast-like cells (bOBs) were isolated from the cranial base of postnatal day 4 mouse pups (Fig. 1) as described in Materials and Methods and cultured in 3D organotypic culture as micromasses (MMs). MMs were grown in standard osteogenic medium (OM) in the presence or absence of BMP6 supplementation for up to 6 weeks. While there was substantial variation in the total cell yield across experiments, there was steady growth from passage 0 to 2 in each experiment (Fig. 2A). The morphology of cells obtained by partial matrix digestion and outgrowth methods was typical of an osteoblast (Fig. 2B), although an occasional osteocyte was observed in earlier passages. Over the course of MM culture, distinct differences became apparent between treatment groups. Morphological changes were evident in OM as early as week 1 of induction, as cells on the top layer of MMs adopted a rounded morphology (Fig. 3, week 1, white arrowheads). Rounded cells were present throughout the remainder of the culture period, and small clusters of rounded cells that had detached from the MM were frequently observed floating in the culture medium microscopically. Tissue condensations, or nodules, were first detected in control OM MMs at week 3. These nodules increased in number and appeared to increase in size throughout the final 3 weeks of culture (Fig. 3, yellow arrowheads). In MMs treated with BMP6 medium, substantial phenotypic changes were evident by week 2, as a subset of cells displayed an enlarged, block-like morphology. These cells were particularly evident at the periphery of MMs (Fig. 3, week 2 inset, red ovals), but were also present in the central region. Extracellular matrix deposition was readily visible in BMP6-treated MMs by week 2, and increased in density through week 6. Early on, matrix accumulation was particularly prominent in the pericellular region of cells with a hypertrophic phenotype (Fig. 3, week 2, blue arrowheads).

### Evaluation of matrix markers

**Figure 4:**
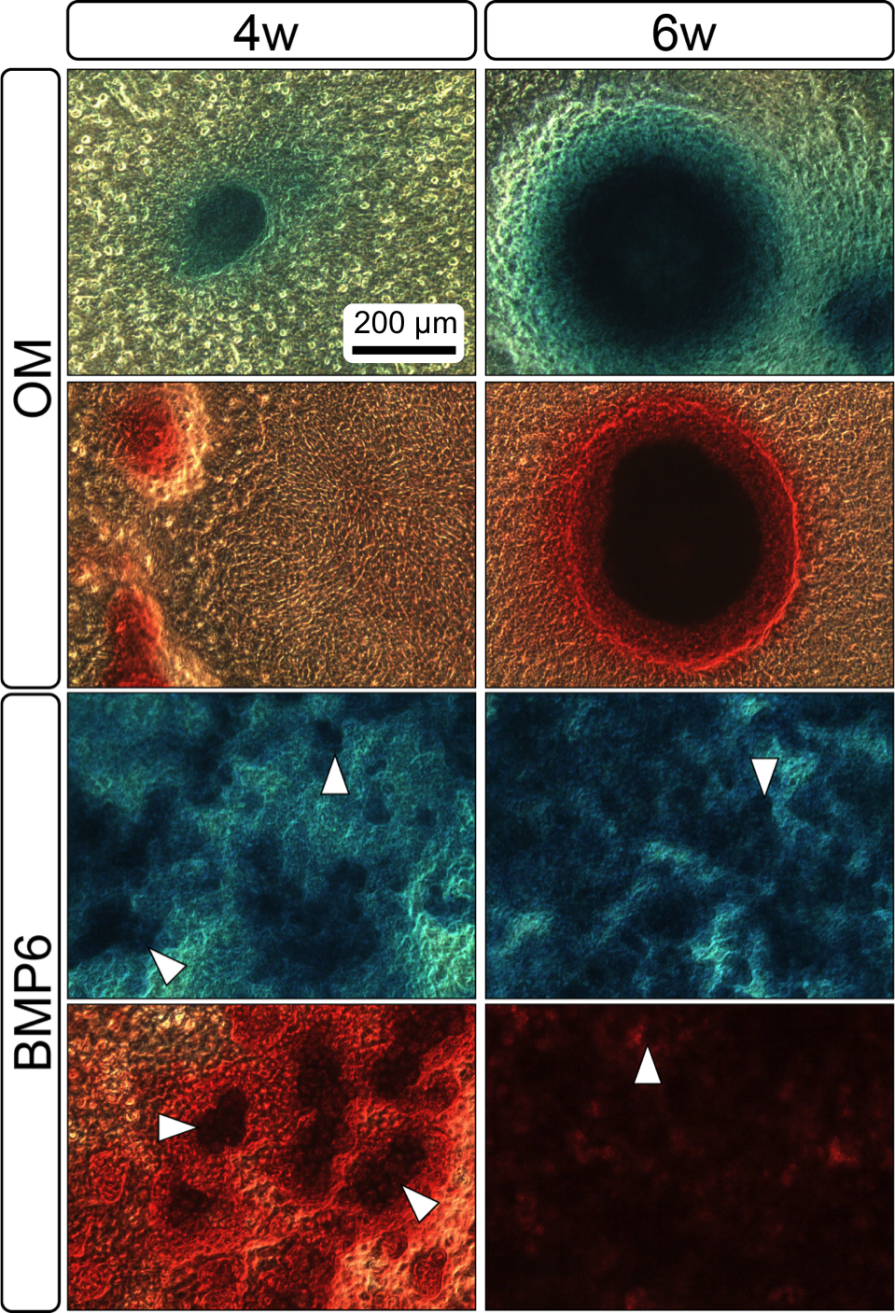
Staining for chondrogenic and osteogenic matrix markers. MMs were stained at 4 and 6 weeks (4w, 6w, respectively) with Alcian Blue to identify accumulation of sulfated glycosaminoglycans (sGAGs), a marker for chondrogenic matrix, and Alizarin Red S to highlight calcium-rich matrix, an indicator of osteogenic tissue. Note that nodules in OM cultures stained for both Alcian Blue and Alizarin Red. In BMP6 cultures, the strongest staining was observed in the pericellular space around enlarged cells with a hypertrophic morphology (white arrowheads). Both matrix density and stain intensity appeared to increase between weeks 4 and 6 in BMP6 cultures. Scale: 200 µm for all images.

Both 4- and 6-week MMs stained extensively with Alcian Blue in the BMP6-treated group, indicating the presence of sulfated glycosaminoglycans (sGAGs), a chondrogenic matrix marker. Notably, 4- and 6-week BMP6-treated cultures also stained strongly for Alizarin Red S, demonstrating a calcium-rich mineralized matrix. For both stains, matrix accumulation was most dense in the central region of the MM, and stain intensity decreased with increasing distance from the center. The most intense staining was typically observed in the pericellular region of individual cells (white arrowheads, Fig. 4), although more diffuse staining was also observed in the surrounding matrix. This trend was less clear at 6 weeks due to the dense nature of the tissue. In OM controls without BMP6, some positive staining for both Alcian Blue and Alizarin Red was evident throughout MMs at both 4 and 6 weeks, though to a lesser extent than in BMP6-treated MMs. There was some light, diffuse staining in the central MM matrix; however, staining for both markers was most intense at discrete regions in which cells had condensed to form small tissue nodules.

### Characterization of growth and differentiation properties

**Figure 5:**
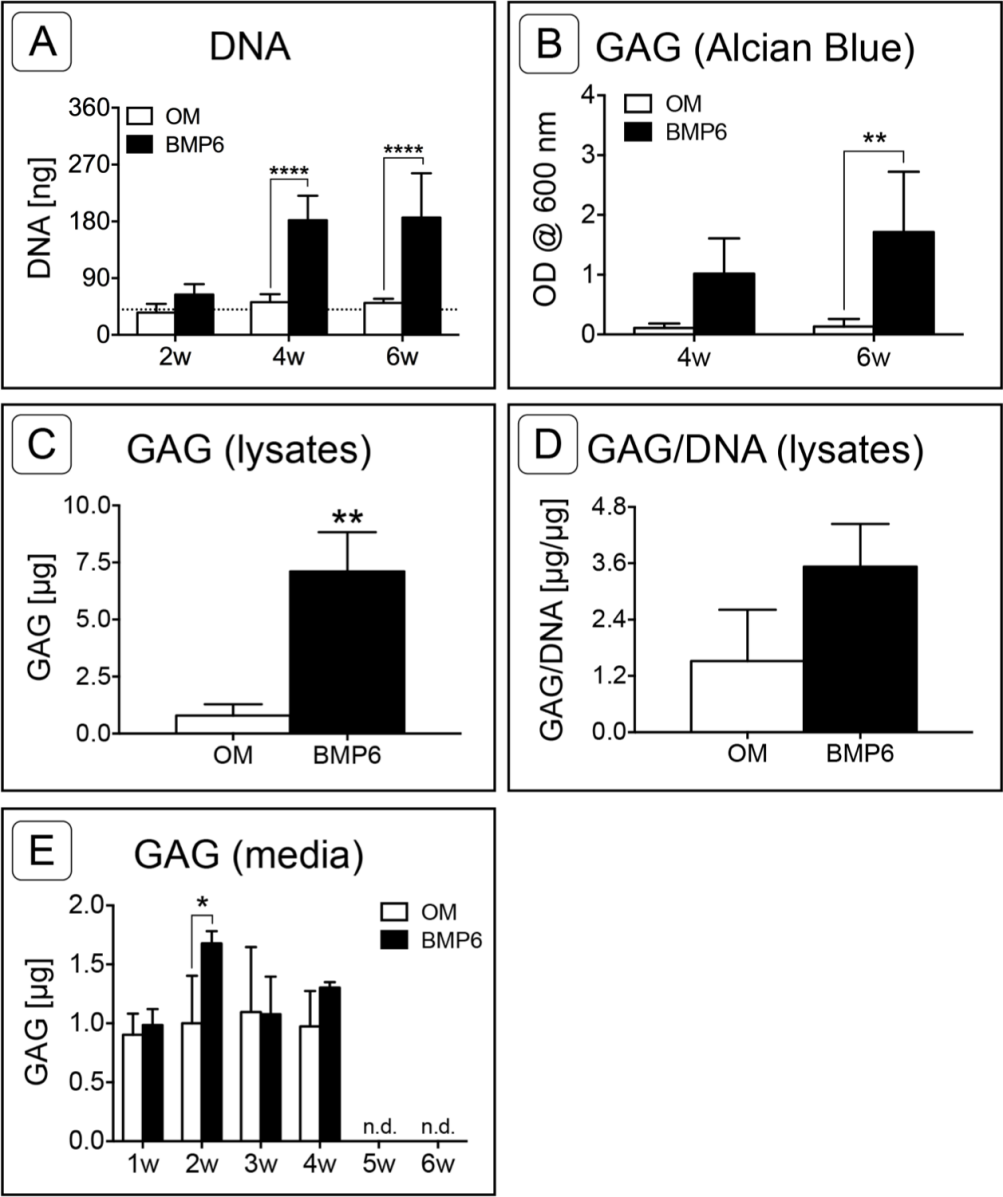
Quantitative assessment of DNA and glycosaminoglycan content. (A) DNA content was evaluated at 2, 4 and 6 weeks (2w, 4w, 6w) in two replicate experiments. Two-way ANOVA and Sidak’s post-test, *n* = 6. The dotted line indicates the average starting DNA content of d0 samples. (B–E) sGAG content was quantified from solubilized Alcian blue dye (B), in 6-week cell lysates (C, D), and from conditioned culture medium (E). (B) Dye from 4- and 6-week Alcian Blue-stained MMs was solubilized and the absorbance was measured at 600 nm. Two replicate experiments, two-way ANOVA and Sidak’s post-test, *n* = 4. (C) Alcian Blue results were verified by quantifying the sGAG content of 6-week MM lysates from 1 experiment. Unpaired *t*-test, *n* = 3. (D) The average sGAG content per cell was quantified by normalizing measured sGAG to sample-matched DNA content. Unpaired *t*-test, *n* = 3. (E) sGAG released into the culture medium was quantified from media samples that were collected at weekly intervals. Two-way ANOVA and Sidak’s post-test, *n* = 3. ‘n.d.’ indicates ‘not detected.’ For all graphs, significance is denoted as follows: *: *p* < 0.05, **: *p* < 0.01, ****: *p* < 0.0001.

DNA was quantified at 2-week intervals as a measure of cell count in the MMs over time. The mean DNA content of MMs grown in control OM did not change over the course of the study and remained consistent with day 0 levels. Conversely, an increase in the DNA content of BMP6-treated MMs was observed between 2 and 4 weeks, resulting in significantly higher DNA levels relative to that of control OM MMs at weeks 4 and 6 (Fig. 5A; *n* = 6, *p* < 0.0001).

Sulfated glycosaminoglycan content was assessed in Alcian Blue stained MMs via solubilization of dye at 4 and 6 weeks or by quantifying the sGAG content of 6-week cell lysates. Since sGAG can be released from the matrix into the culture medium (Steward, Wagner & Kelly, 2013), sGAG content was also quantified from conditioned culture medium samples taken at weekly time points from weeks 1 through 6 of induction. Quantification of solubilized Alcian Blue dye at 4 and 6 weeks demonstrated that BMP6 treatment resulted in significantly more accumulation of sGAGs than control OM treatment (Fig. 5B; *n* = 4, *p* < 0.01). Similarly, there was significantly more sGAG content in BMP6-treated MM lysates at the 6-week time point than in control OM samples (Fig. 5C; *n* = 3, *p* < 0.01). However, when normalized to the DNA content of matched samples, the average sGAG per cell was not significantly different between groups (Fig. 5D; *n* = 3). While BMP6 treatment (*p* < 0.05) and time point (*p* < 0.0001) were significant factors in the sGAG released into media samples, the secreted sGAG measured at a given week was only significantly different between control OM and BMP6-treated samples at 2 weeks (*p* < 0.05), and no sGAG was detected in 5- and 6-week samples (Fig. 5E; *n* = 3).

**Figure 6:**
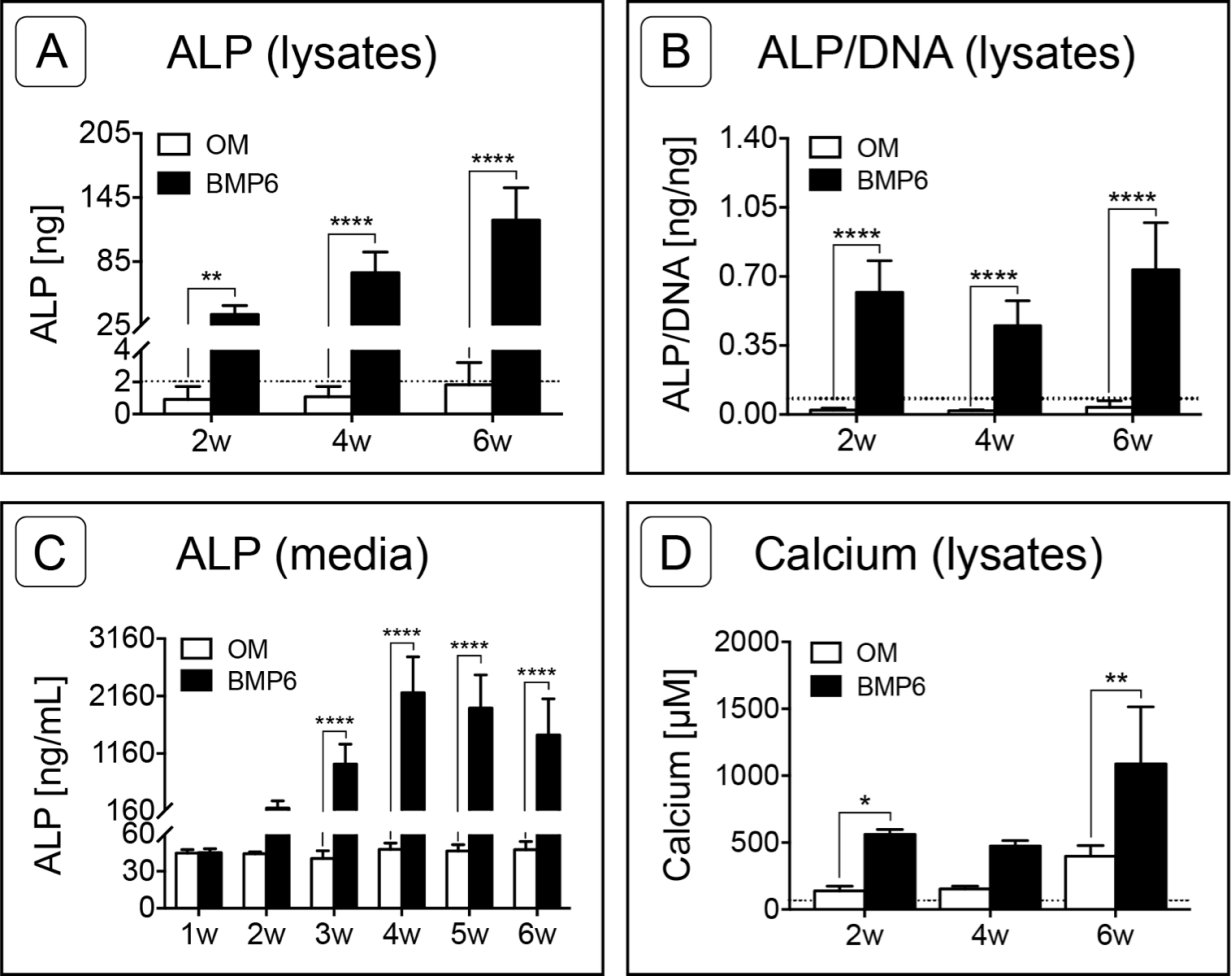
Quantification of osteogenic markers. (A) Alkaline Phosphatase (ALP) was quantified from MM lysates of 2 replicate experiments at weeks 2, 4 and 6 (2w, 4w, 6w, respectively), *n* = 6. (B) Average ALP activity per cell was assessed by normalizing ALP content of lysates to sample-matched DNA content, two replicate experiments, *n* = 6. (C) ALP released into the medium was quantified from samples taken at weekly intervals from weeks 1–6, two replicate experiments, *n* = 6. (D) Calcium content of MM lysates at weeks 2, 4 and 6, one experiment, *n* = 3. For all graphs, significance was assessed via two-way ANOVA and Sidak’s post-test: *: *p* < 0.05, **: *p* < 0.01, ****: *p* < 0.001. Dotted lines in A, B and D represent the average d0 value for the given marker.

Alkaline Phosphatase (ALP) content was measured in 0-, 2-, 4- and 6-week cell lysates as well as from weekly media samples collected from weeks 1 through 6. As assessed by lysates, ALP content increased in BMP6-treated MMs at each successive 2-week interval relative to d0 reference samples, while ALP in control OM cultures remained relatively constant over the 6-week induction period (Fig. 6A; *n* = 6; *p* < 0.01). ALP levels of BMP6-treated samples were significantly higher than those of OM samples at all 3 time points (*n* = 6, 2w: *p* < 0.01, 4w and 6w: *p* < 0.0001). Likewise, the average ALP per cell, determined by normalizing the ALP content of each sample to its DNA content, was significantly higher in the BMP6-treated group at weeks 2, 4 and 6 (Fig. 6B; *n* = 6, *p* < 0.0001). A similar trend was observed in conditioned media samples, as ALP content was significantly higher in BMP6-treated samples than in control OM samples from weeks 3 through 6 (Fig. 6C; *n* = 6, *p* < 0.0001). Consistent with cell lysates, the ALP content of control OM media samples remained unchanged over the 6 weeks of culture (Fig. 6C).

Calcium content was quantified from MM lysates at 2-week intervals beginning at d0. Similar to ALP content, calcium was significantly higher in BMP6-treated MMs compared to OM controls at weeks 2 (*p* < 0.05) and 6 (*p* < 0.01), but not at week 4 (Fig. 6D, *n* = 3). Interestingly, both conditions displayed the same trend over the time points evaluated, in which the mean calcium content of MMs remained relatively consistent from weeks 2 to 4, followed by an increase from week 4 to week 6.

### Expression of candidate genes

To further characterize the osteogenic potential of basicranial osteoblasts, the expression of key chondrogenic, proliferative, hypertrophic and osteogenic genes was assessed at 4 and 6 weeks of induction. Fold-induction for the genes of interest was calculated using the 2^−ΔΔ*CT*^ method, with *GAPDH* as the housekeeping gene and day 0 as the reference time point. Expression of day 0 values was also assessed via the 2^−Δ*CT*^ method to aid in characterization of the phenotype present at the start of induction.

**Figure 7:**
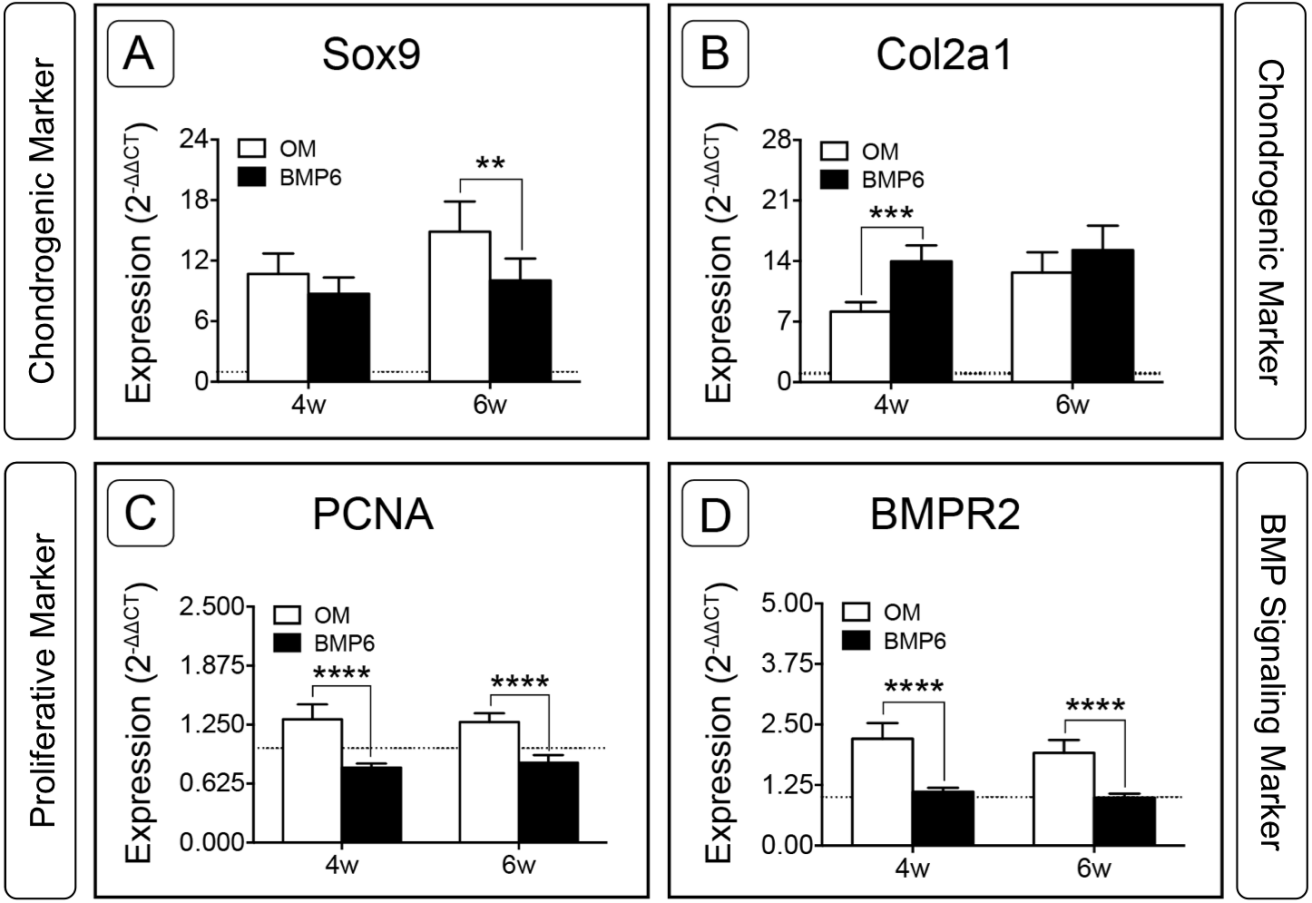
Expression of chondrogenic, proliferative and BMP signaling pathway genes in micromass cultures. Chondrogenic markers *Sox9* (A) and *Col2a1* (B), proliferative marker *PCNA* (C) and BMP signaling pathway marker *BMPR2* (D) were evaluated at 4 and 6 weeks of treatment (4w, 6w) from 2 replicate experiments, *n* = 6. Fold-induction was calculated using the 2^−ΔΔ*CT*^ method with day 0 expression as a reference condition. The dotted line is set to *y* = 1.0, representing the threshold for no change in expression relative to day 0. For all graphs, significance was assessed via two-way ANOVA and Sidak’s post-test: **: *p* < 0.01, ***: *p* < 0.001, ****: *p* < 0.0001.

For the two chondrogenic genes evaluated, BMP6 treatment resulted in distinct trends. Transcription factor SRY (Sex-Determining Region Y)-Box 9 (*Sox9*) increased in both treatment groups and at both time points relative to d0, but was significantly down-regulated in BMP6-treated MMs compared to OM controls at 6 weeks (Fig. 7A; *n* = 6, *p* < 0.005). Type II collagen (*Col2a1*) expression in all groups was also higher than the d0 level, but was significantly up-regulated with BMP6 treatment at 4 weeks compared to control OM (Fig. 7B; *n* = 6, *p* < 0.001).

Proliferating Cell Nuclear Antigen (*PCNA*) was investigated to evaluate the proliferative response of bOBs. *PCNA* expression increased in the OM control group and decreased in the BMP6-treated group relative to the d0 reference, and BMP6 treatment resulted in significant down-regulation compared to control OM at both 4 and 6 weeks (Fig. 7C; *n* = 6, *p* < 0.0001). A similar trend was observed for BMP signaling pathway marker Bone Morphogenetic Protein Receptor Type II (*BMPR2*) expression, which was significantly higher in control OM than BMP6-treated samples at both time points (Fig. 7D; *n* = 6, *p* < 0.0001). OM resulted in increased *BMPR2* expression compared to the d0 reference value, while BMP6 treatment did not induce any deviation from d0 expression levels.

**Figure 8:**
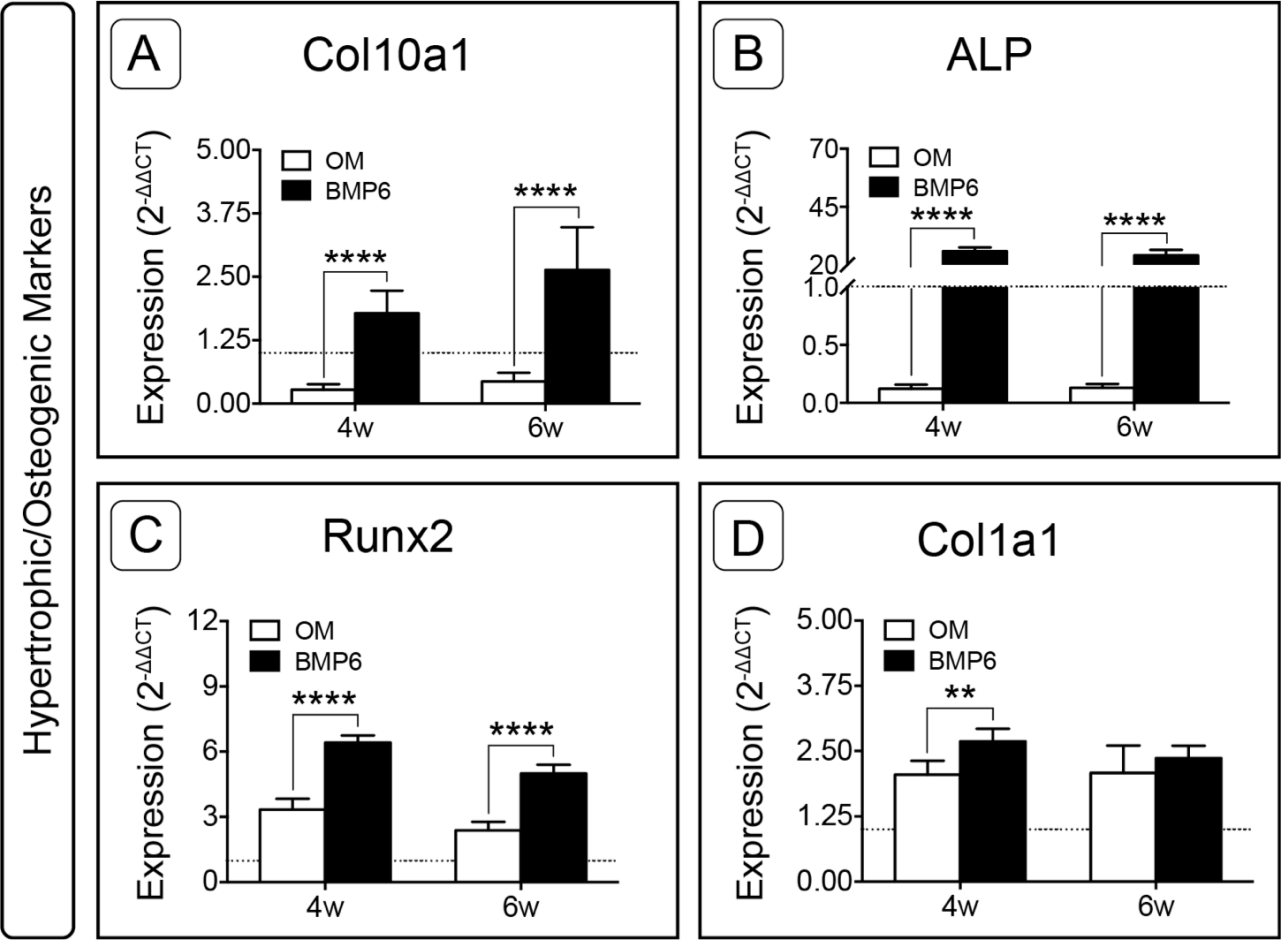
Expression of hypertrophic and osteogenic genes in micromass cultures. Hypertrophic marker *Col10a1* (A) and osteogenic markers *ALP* (B), *Runx2* (C) and *Col1a1* (D) were evaluated at 4 and 6 weeks (4w, 6w). Fold-induction was calculated using the 2^−ΔΔ*CT*^ method. The dotted line at *y* = 1.0 represents the threshold for no change in expression relative to day 0. For all graphs, significance was assessed via two-way ANOVA and Sidak’s post-test, *n* = 6, **: *p* < 0.01, ****: *p* < 0.0001.

Hypertrophic and osteogenic markers were also examined. Following expansion, the only gene of interest that was up-regulated was type I collagen (*Col1a1,* Fig. S1). During induction, BMP6 treatment resulted in up-regulation of all four hypertrophic and osteogenic markers (*Col10a1*, *ALP*, *Runx2*, *Col1a1*) at 4 weeks and three of four hypertrophic/osteogenic markers at 6 weeks when compared to control OM treatment (Fig. 8). This trend was most dramatic for Alkaline Phosphatase, as *ALP* expression in BMP6-treated MMs was vastly up-regulated compared to OM controls at both time points (Fig. 8B; *n* = 6, *p* < 0.0001). Relative to the d0 reference level, *ALP* was lower in OM and higher in BMP6 samples at 4 and 6 weeks. Similarly, type X collagen (*Col10a1*) was significantly up-regulated with BMP6 treatment (*n* = 6, *p* < 0.0001), and compared to d0 samples expression decreased in control OM MMs and increased in BMP6-treated MMs (Fig. 8A). For runt-related transcription factor 2 (*Runx2*), BMP6 treatment resulted in significant up-regulation compared to OM treatment at both time points (*n* = 6, *p* < 0.0001). Unlike *ALP* and *Col10a1*, however, both BMP6 and OM treatments resulted in an increase in *Runx2* compared to the d0 reference (Fig. 8C). Matrix marker type I collagen was significantly up-regulated in BMP6-treated MMs at 4 weeks compared to OM controls (*n* = 6, *p* < 0.01), and both treatments resulted in increased expression relative to d0 levels (Fig. 8D).

## Discussion

Despite its critical role in coordinated craniofacial development and morphogenesis, early bone formation in the basicranium, particularly on a cellular level, remains largely unstudied. This is in contrast to activity at the cartilaginous synchondroses, which has been well documented (Madeline & Elster, 1995; Okamoto et al., 1996; Nie, 2005; Kettunen et al., 2006). While these growth fronts are critical to the lengthening process from embryonic stages to adolescence (Laurita et al., 2011; Som & Naidich, 2013), surprisingly little is known about the hard tissues comprising the majority of the cranial base. In an effort to fill this significant gap, our study demonstrates for the first time the isolation of osteoblast-like cells from the basicranium of neonatal mice, and details the proliferative and mineralization potential of these cells.

### BMP6 treatment initiates a chondrogenic response in bOBs

Osteoblast-like cells were successfully isolated from the basicranium of neonatal mice by a combination of enzymatic digestion and outgrowth methods, and were then expanded and seeded in 3D MM culture. During 2D expansion, cells presented a morphology similar to calvarial osteoblasts (Bakker & Klein-Nulend, 2012) and *Col1a1* was up-regulated, indicating a post-proliferative, pre-mineralizing osteoblast phenotype (Stein, Lian & Owen, 1990; Aubin et al., 1995), or possibly a dedifferentiated chondrocyte phenotype (Lefebvre et al., 1994) at the start of induction. Upon initiation of MM culture cells adopted a rounded phenotype, and with BMP6 treatment a subset of cells became enlarged, morphologically resembling hypertrophic chondrocytes (Mackie et al., 2008). While sparse, discrete nodules of matrix accumulation were observed in MMs treated with standard OM, BMP6 treatment induced rapid matrix deposition throughout MMs. Accumulating matrix stained positive for Alcian Blue, and sGAG content was higher in conditioned culture media samples at 2 weeks and in lysates at 6 weeks. Alcian Blue-positive matrix was strongest in the pericellular region of rounded cells, resembling that of murine chondrocytes cultured in the presence of ascorbic acid (Lefebvre et al., 1994). Moreover, the developing GAG-rich matrix was accompanied by up-regulation of chondrocyte markers *Sox9* and *Col2a1*, further indicating that BMP6 induced a chondrogenic response.

Interestingly, control OM treatment also induced up-regulation of *Sox9* and *Col2a1* relative to d0 levels, but resulted in minimal accumulation of sGAG-rich matrix as assessed by intra- and extracellular measures. This suggests that the MM culture method may be responsible for the observed up-regulation of *Sox9* and *Col2a1*, rather than being a result of BMP6 treatment. This assessment seems even more likely considering that the induction media are comprised of the same components as growth medium, with the addition of *β*-glycerophosphate to each, and also BMP6 to treatment medium.

### Continued BMP6 treatment induces up-regulation of markers associated with hypertrophy and/or osteoblasts

Following a predominantly chondrogenic response, an increase in markers associated with hypertrophy and early osteogenesis was observed in the BMP6 group. Peak ALP activity was detected in media samples at 4 weeks of induction, after which high levels of secreted and intracellular ALP were maintained. High ALP content coincided with the loss of detectable GAG content in media samples during the final 2 weeks of induction, and an increase in intracellular calcium content at 6 weeks, possibly indicating a phenotypic transition. In accordance with biochemical analyses, expression of hypertrophic marker *Col10a1* and hypertrophic/osteogenic markers *ALP* and *Runx2* were significantly up-regulated with BMP6 treatment relative to both OM and d0 levels at 4 and 6 weeks. These findings are consistent with progression to the late stages of hypertrophy in which cells stop producing cartilaginous matrix, begin to express *Col10a1* and makers associated with osteoblasts, such as *ALP*, and direct tissue mineralization in preparation for subsequent bone formation (Gerstenfeld & Shapiro, 1996; Lefebvre & Smits, 2005). Given its suspected role in late stage chondrocyte differentiation (Ebisawa et al., 1999; Kettunen et al., 2006), we suggest that BMP6 is responsible for the observed increase in *Col10a1* expression.

### Expression of chondrogenic, hypertrophic and osteogenic genes indicates potential heterogeneity in the isolated cell population

In general, expression of the evaluated genes changed very little between 4 and 6 weeks for the BMP6-treated group; however, certain trends became clear when comparing BMP6 expression to that of the control OM group. The strongest trends were observed for proliferative (*PCNA*) and BMP signaling pathway (*BMPR2*) markers, which were significantly lower for BMP6-treated MMs at both time points, and for hypertrophic/osteogenic markers *Col10a1*, *ALP* and *Runx2*, which were significantly higher for the BMP6-treated group at 4 and 6 weeks. This agrees with measures of DNA content, which demonstrated an early proliferative response between weeks 2 and 4 via increased DNA levels compared to d0 and OM values, after which DNA content remained unchanged between weeks 4 and 6. Together, these findings indicate that cells were committed to differentiation and matrix production rather than proliferation at 4 and 6 weeks (Stein, Lian & Owen, 1990). Surprisingly, *BMPR2* expression tracked with proliferative marker *PCNA* rather than with osteogenic markers. It may be that 4- and 6-week time points were too late to detect changes in expression patterns. This would be consistent with the findings of an ectopic bone formation study in which *BMPR2* expression peaked at 4 days post-implantation in both BMP2-positive and–negative samples (Nakamura et al., 2003). Similarly, *BMPR2* was constitutively expressed in calvarial osteoblasts, but showed no significant changes in expression at 7 and 17 days of osteogenic differentiation *in vitro* (Kalajzic et al., 2005). This suggests that evaluation of *BMPR2* expression levels at time points earlier than 1 week may be beneficial to future research.

A similar sequence of events to that observed in the current study (proliferation; adoption of a rounded phenotype; GAG-rich matrix deposition; and mineralization) was previously reported for chick hypertrophic chondrocytes that were cultured in the presence of ascorbic acid (Descalzi Cancedda et al., 1992). Considering these data alone, one might conclude that the isolated cell population was comprised of hypertrophic chondrocytes (Descalzi Cancedda et al., 1992; Lefebvre & Smits, 2005; Yang et al., 2014). However, in addition to elevated *Col10a1* expression, chondrogenic marker *Col2a1* and osteogenic marker *Col1a1* were both up-regulated with BMP6 treatment at 4 weeks. Additionally, expression of these markers was higher than d0 levels for both groups at 4 and 6 weeks, raising questions as to the nature of the isolated cells.

Since *Col2a1* is expressed in chondrocytes and pre-hypertrophic chondrocytes, but not osteoblasts (Alborzi et al., 1996; Lefebvre & Smits, 2005), and *Col1a1* is expressed in osteoblasts, but not in mammalian chondrocytes or hypertrophic chondrocytes (Aubin et al., 1995; Yang et al., 2014), we posit that this apparent contradiction may be explained by a heterogeneous population of cells within the micromass. Increased *Col1a1* and *Runx2* expression in both treatment groups could be attributed to a population of osteoblasts, while a population of chondrocytes would account for elevated *Sox9* and *Col2a1* expression in all samples compared to d0. Under certain culture conditions, *Col1a1*-expressing dedifferentiated chondrocytes are able to regain their chondrogenic phenotype (Cancedda et al., 2000), which may have been possible with the transition from monolayer expansion to a micromass culture system, and would thus explain up-regulation of chondrogenic markers in the micromass during induction. Finally, the presence of hypertrophic chondrocytes would account for the significant up-regulation in *Col10a1* expression in BMP6-treated cultures, and may have also contributed to elevated levels of *ALP* and *Runx2* along with ALP and calcium content. Notably, this effect was absent in the control OM group, as *Col10a1* and *ALP* expression were lower than d0 levels, and ALP and calcium content remained relatively constant throughout the induction period. These results are consistent with observed differences in morphology between the two induction media, and support our previous assertion that BMP6 treatment induced a hypertrophic response in a subset of the isolated cells.

### Potential extrinsic and intrinsic causes of heterogeneity in mammalian bOBs

Together, the results of this study demonstrate that BMP6 treatment is a potent inducer of mineralization via an endochondral-like pathway in neonatal mammalian basicranial-derived cells *in vitro,* while standard OM is ineffective in stimulating mineralization. This is in contrast to previous studies, which have demonstrated that bone cells derived from various skeletal sites including the calvarium (Ecarot-Charrier et al., 1983; Ecarot-Charrier et al., 1988; Moursi et al., 1996; Quarto et al., 2010), mandible (Cooper et al., 1998) and long bones (Stringa et al., 1995; Collignon, Davicco & Barlet, 1997), as well as hypertrophic chondrocytes in the limbs (Descalzi Cancedda et al., 1992; Kirsch et al., 1997) and osteoblastic MC3T3 cells (Quarles et al., 1992; Lian et al., 1997; Wang et al., 1999), mineralize in standard OM lacking BMP6. Thus, more direct comparisons are needed to determine the extent to which bOBs are distinct from other cranial osteoblasts. Interestingly, biochemical and gene expression analyses revealed that the isolated cells were heterogeneous, containing populations of both osteogenic and chondrogenic lineages despite the fact that synchondroses were removed prior to cell isolation.

The persistence of a chondrogenic phenotype apart from the spheno-occipital synchondrosis is surprising since the neonatal cranial base is well mineralized by stage P3, as visualized by Alizarin Red staining (Shum et al., 2003), and vasculature and bone marrow cavities are evident in the trabecular bone of the basisphenoid and basioccipital in newborn mice (Kettunen et al., 2006). It is possible that the observed phenotypic heterogeneity may be related to the endochondral origins of the cranial base, which would suggest that ossification mode is an important determinant of cell behavior in the neonatal basicranium of mammals. However, given the disparity in mineralization capacity compared to prior work on osteoblasts in long bones, which are also endochondrally-derived, our findings suggest that ossification mode alone cannot fully explain the unique characteristics of bOBs.

One noteworthy distinction between the cranial base and limbs is their mechanical loading *in vivo*, which differs in both magnitude and pattern. Long bones are subjected to high-magnitude cyclic loads during locomotion (Rubin & Lanyon, 1984). In contrast, the predominate loads experienced by basicranial bone are low-magnitude static loads related to supporting the brain (Enlow, 1990; Richtsmeier & Flaherty, 2013). Indeed, bones of the cranial vault are minimally influenced by masticatory stresses (Ravosa, Johnson & Hylander, 2000; Ravosa et al., 2010; Franks et al., 2016; Franks et al., 2017).

The functional loading environment of bones has been implicated in contrasting responses of limb and calvarial cells to applied mechanical loads *in vitro* (Rawlinson et al., 1995) and it follows that such a relationship may also extend to the lower portion of the braincase. For instance, indirect, cyclic mechanical stimuli applied to the basicranium via loading of the maxillae or premaxillae induced chondrocyte proliferation and increased tissue volume within the spheno-occipital synchondrosis in rabbits (Wang & Mao, 2002a; Wang & Mao, 2002b), indicating that mechanical loads affect chondral growth of the cranial base. Although strain was not applied to micromass cultures in the present study, prior findings (Rawlinson et al., 1995; Wang & Mao, 2002a; Wang & Mao, 2002b) suggest that the strain environment of the cranial base is an important consideration in understanding its growth and osteogenic potential.

In addition to ossification mode and loading pattern, cranial elements vary with respect to embryological origin. The basicranium, and the isolated cell population, is comprised of both neural crest and mesoderm-derived cells (Som & Naidich, 2013). This complicates interpretation of the osteogenic potential of basicranial cells as it relates to embryology; however, previous studies may provide some insight. In monolayer culture, frontal cells of neural crest origin treated with OM exhibited enhanced osteogenic potential relative to mesodermal parietal cells (Quarto et al., 2010), suggesting that embryology influences cranial cell behavior. Despite their lesser potential, parietal cells produced small mineral nodules throughout the cultures under standard osteogenic medium treatment (Quarto et al., 2010). Similarly, a few mineralized nodules were observed in control OM-treated basicranial micromasses, possibly indicating that mesoderm-derived cells dominated the mineralization response. This finding may be a function of a neural crest migration, in which mesodermal cells progressively invade the spheno-occipital synchondrosis and basisphenoid bone postnatally (McBratney-Owen et al., 2008). Enhanced chondrogenesis and mineralization related to BMP6 treatment may likewise be driven by a mesodermal response. This is reflected in BMP6 expression patterns in the developing cranial base, as BMP6 transcripts were detected in the mesenchymal condensation of the basioccipital bone, but not in the neural crest-derived basisphenoid condensation (Kettunen et al., 2006). It is unlikely, however, that embryology is a dominant factor in driving basicranial OB behavior because they produced markedly less mineralization in response to OM than calvarial cells of both origins under similar culture conditions (i.e., parietal and, especially, frontal bones; Quarto et al., 2010), indicating an even further diminished osteogenic potential.

## Summary and Conclusion

Despite the fact that it is the oldest component of the vertebrate skull (De Beer, 1937), much remains unknown about basicranial development. The cranial base plays a critical role in the coordinated growth, development and evolution of the cranium and brain, and provides passage for cranial nerves to connect with the rest of the body (Lieberman, Ross & Ravosa, 2000; Richtsmeier & Flaherty, 2013). As such, it has a unique set of characteristics that distinguish it from all other skeletal sites, and has been described as “the most complex structure of the skeleton” (Nie, 2005). It is therefore unsurprising that the cranial base has garnered much interest from biologists and anthropologists alike. However, much of the research on the basicranium has focused on its morphological characteristics from both phylogenetic and ontogenetic perspectives (Ross & Ravosa, 1993; Lieberman, Ross & Ravosa, 2000; Jeffery, 2003; Shum et al., 2003; Nie, 2005; López et al., 2008).

To this end, we explored the distinct nature of the developing basicranium on a cellular level by evaluating the osteogenic potential of non-synchondrosal cells cultured in 3D micromasses. The results of our study indicate that BMP6 treatment is required to induce mineralization in basicranial osteoblast-like cells derived from neonatal mice. The lack of mineral accumulation in response to treatment with OM suggests that heterogeneity exists among cranial elements and, more broadly, that these cells are unique compared to osteoblasts derived from other sites within the skull and postcranial skeleton. Considering these interesting differences in the context of cranial base development, we propose that ossification mode, functional loading environment, and possibly embryology may be determinants of the distinct osteogenic potential of basicranial osteoblasts. Such findings have implications for understanding cranial base development on a fundamental level, as well as for identifying the mechanistic basis of morphological transformations during craniofacial evolution. This underscores the importance of a multi-scale analysis that considers morphological characteristics, architectural properties *and* cellular behavior. Expanding upon the results presented herein to further elucidate the behavior of cranial base-derived cells throughout ontogeny is also critical to optimizing corrective treatments for craniofacial disorders that seek to stimulate basicranial growth. Specifically, characterizing the basicranial cell population at the time of isolation and evaluating additional markers such as Indian Hedgehog (Ihh) and Parathyroid Hormone (PTH) during induction would provide important insight into the chondrogenic and mineralization responses observed in the current study. Indeed, the apparent site-specific uniqueness of basicranial osteoblasts suggests that intrinsic heterogeneity in osteoblast behavior may play a greater role in determining observed patterns of phenotypic variation throughout the developing skeleton.

##  Supplemental Information

10.7717/peerj.5757/supp-1 Supplemental Information 1 Expression of candidate genes following expansion Each bar represents the average expression of a particular candidate gene following expansion of basicranial osteoblasts and prior to induction. Data are represented as 2^−*dCT*^ relative to housekeeping gene GAPDH (*n* = 6). Values above the dotted line (*y* = 1) indicates higher expression than GAPDH, while values below the line indicate diminished expression.

10.7717/peerj.5757/supp-2 Supplemental Information 2 Figure 2 P0 basicranial OBs

10.7717/peerj.5757/supp-3 Supplemental Information 3 Figure 2 P1 basicranial OBs

10.7717/peerj.5757/supp-4 Supplemental Information 4 Figure 2 P2 basicranial OBs

10.7717/peerj.5757/supp-5 Supplemental Information 5 Figure 2 cell counts

10.7717/peerj.5757/supp-6 Supplemental Information 6 Basicranial osteoblast MM with 1 week of BMP6 treatment

10.7717/peerj.5757/supp-7 Supplemental Information 7 Basicranial osteoblast MM with 1 week of OM treatment

10.7717/peerj.5757/supp-8 Supplemental Information 8 Basicranial osteoblast MM with 2 weeks of BMP6 treatment

10.7717/peerj.5757/supp-9 Supplemental Information 9 Basicranial osteoblast MM with 2 weeks of OM treatment

10.7717/peerj.5757/supp-10 Supplemental Information 10 Basicranial osteoblast MM with 3 weeks of OM treatment

10.7717/peerj.5757/supp-11 Supplemental Information 11 Basicranial osteoblast MM with 3 weeks of BMP6 treatment

10.7717/peerj.5757/supp-12 Supplemental Information 12 Basicranial osteoblast MM with 4 weeks of OM treatment

10.7717/peerj.5757/supp-13 Supplemental Information 13 Basicranial osteoblast MM with 4 weeks of BMP6 treatment

10.7717/peerj.5757/supp-14 Supplemental Information 14 Basicranial osteoblast MM with 5 weeks of BMP6 treatment

10.7717/peerj.5757/supp-15 Supplemental Information 15 Basicranial osteoblast MM with 5 weeks of OM treatment

10.7717/peerj.5757/supp-16 Supplemental Information 16 Basicranial osteoblast MM nodule with 6 weeks of OM treatment

10.7717/peerj.5757/supp-17 Supplemental Information 17 Basicranial osteoblast MM with 6 weeks of OM treatment

10.7717/peerj.5757/supp-18 Supplemental Information 18 Basicranial osteoblast MM with 6 weeks of BMP6 treatment

10.7717/peerj.5757/supp-19 Supplemental Information 19 Basicranial osteoblast MM treated for 4 weeks with OM stained with Alcian Blue

10.7717/peerj.5757/supp-20 Supplemental Information 20 Basicranial osteoblast MM treated for 4 weeks with OM stained with Alizarin Red

10.7717/peerj.5757/supp-21 Supplemental Information 21 Basicranial osteoblast MM treated for 4 weeks with BMP6 stained with Alcian Blue

10.7717/peerj.5757/supp-22 Supplemental Information 22 Basicranial osteoblast MM treated for 4 weeks with BMP6 stained with Alizarin Red

10.7717/peerj.5757/supp-23 Supplemental Information 23 Basicranial osteoblast MM treated for 6 weeks with OM stained with Alcian Blue

10.7717/peerj.5757/supp-24 Supplemental Information 24 Basicranial osteoblast MM treated for 6 weeks with BMP6 stained with Alcian Blue

10.7717/peerj.5757/supp-25 Supplemental Information 25 Basicranial osteblast MM with 6 weeks OM treatment stained with Alizarin Red

10.7717/peerj.5757/supp-26 Supplemental Information 26 Basicranial osteoblast MM treated for 6 weeks with BMP6 stained with Alizarin Red

10.7717/peerj.5757/supp-27 Supplemental Information 27 Figure 5 DNA lysate raw data

10.7717/peerj.5757/supp-28 Supplemental Information 28 Figure 5 GAG lysates and media samples

10.7717/peerj.5757/supp-29 Supplemental Information 29 Figure 5 GAG Alcian Blue raw data

10.7717/peerj.5757/supp-30 Supplemental Information 30 Figure 6 ALP and DNA lysate raw data

10.7717/peerj.5757/supp-31 Supplemental Information 31 Figure 6 calcium lysates raw data

10.7717/peerj.5757/supp-32 Supplemental Information 32 Figures 7 & 8 raw CT values

## Additional Information and Declarations

### Data Availability

The following information was supplied regarding data availability: The raw data are provided in the Supplemental Files.
