# Supplementary figures and images for: Non-sutural basicranium-derived cells undergo a unique mineralization pathway via a cartilage intermediate in vitro
Source: PeerJ. 2018 Oct 23;6:e5757. doi: 10.7717/peerj.5757 (PMC6202976; doi:10.7717/peerj.5757)

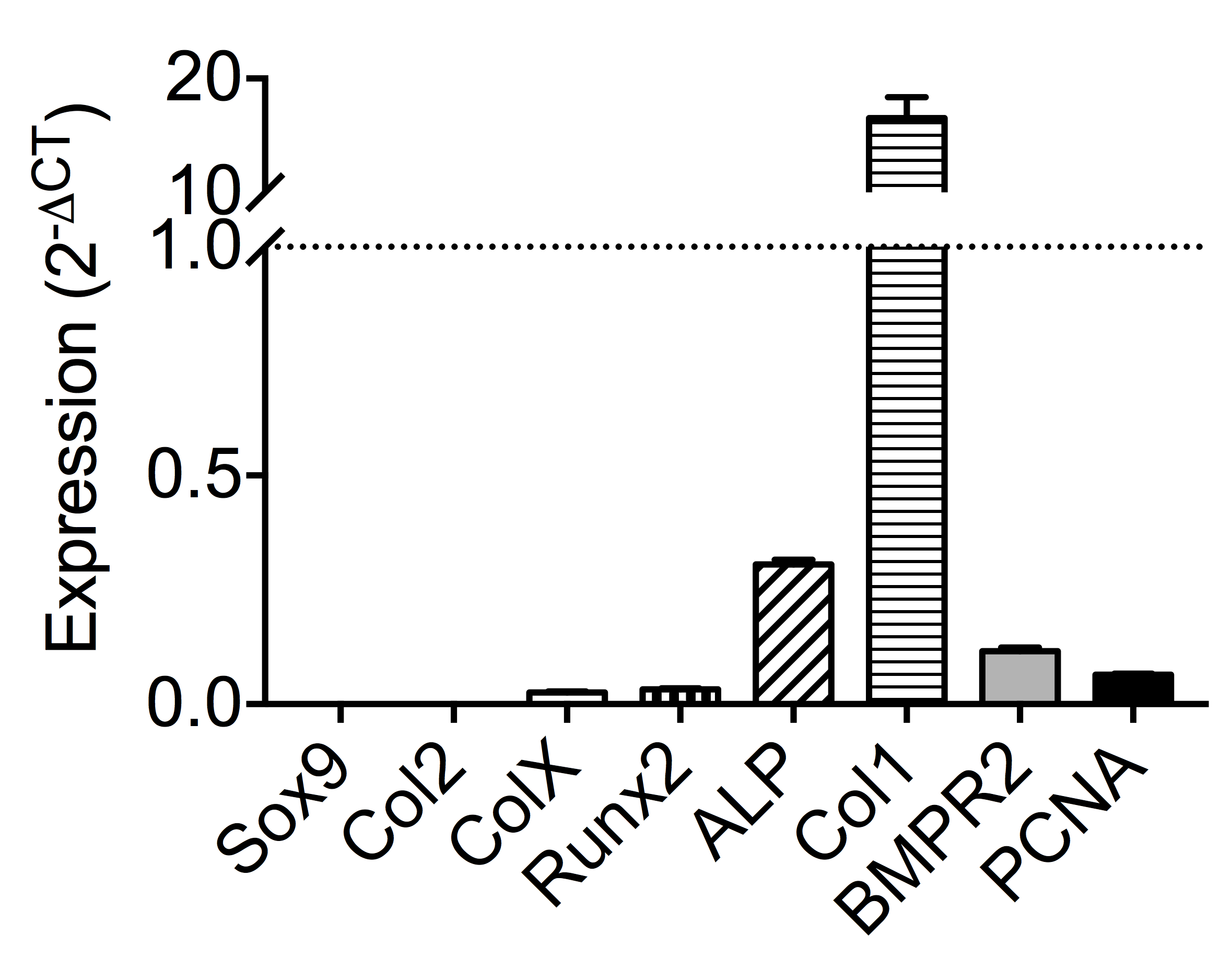

Supplement: Supplemental Information 1 — Each bar represents the average expression of a particular candidate gene following expansion of basicranial osteoblasts and prior to induction. Data are represented as 2−dCT relative to housekeeping gene GAPDH (n = 6). Values above the dotted line (y = 1) indicates higher expression than GAPDH, while values below the line indicate diminished expression. [file peerj-06-5757-s001.png]

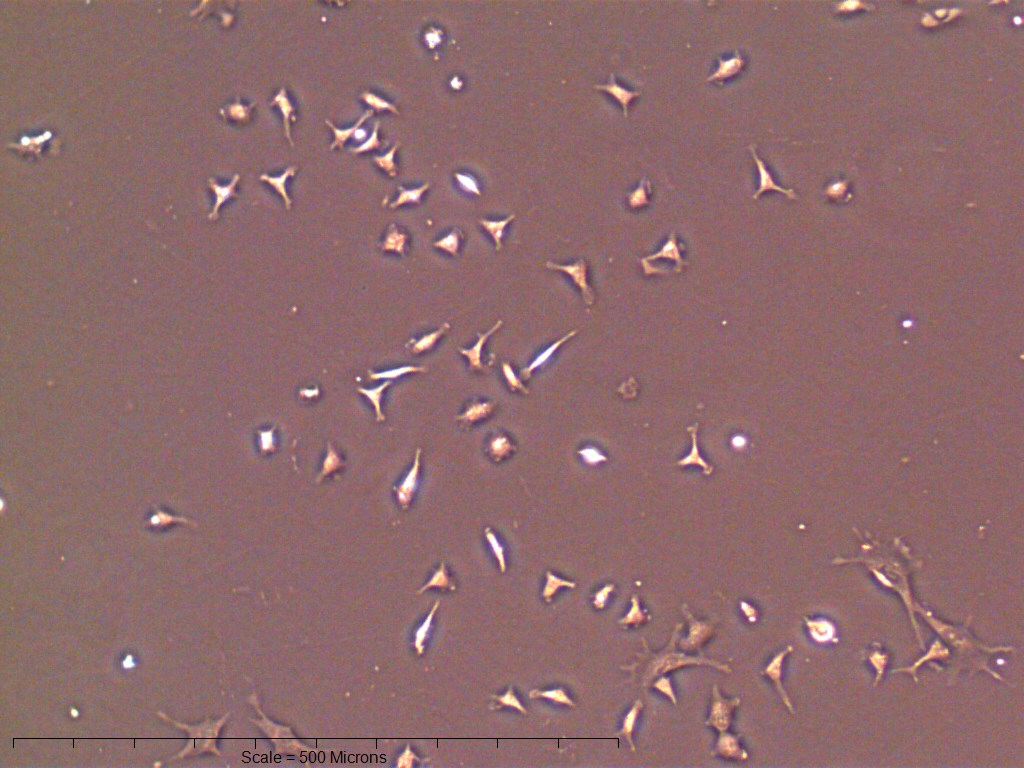

Supplement: Supplemental Information 2 [file peerj-06-5757-s002.jpg]

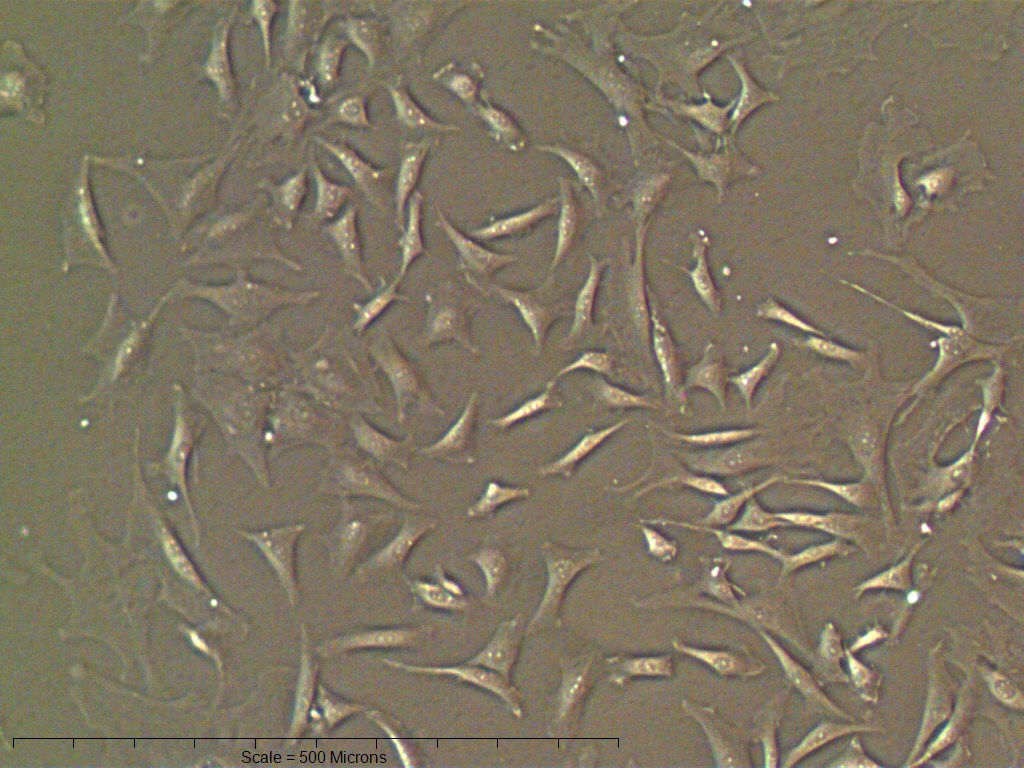

Supplement: Supplemental Information 3 [file peerj-06-5757-s003.jpg]

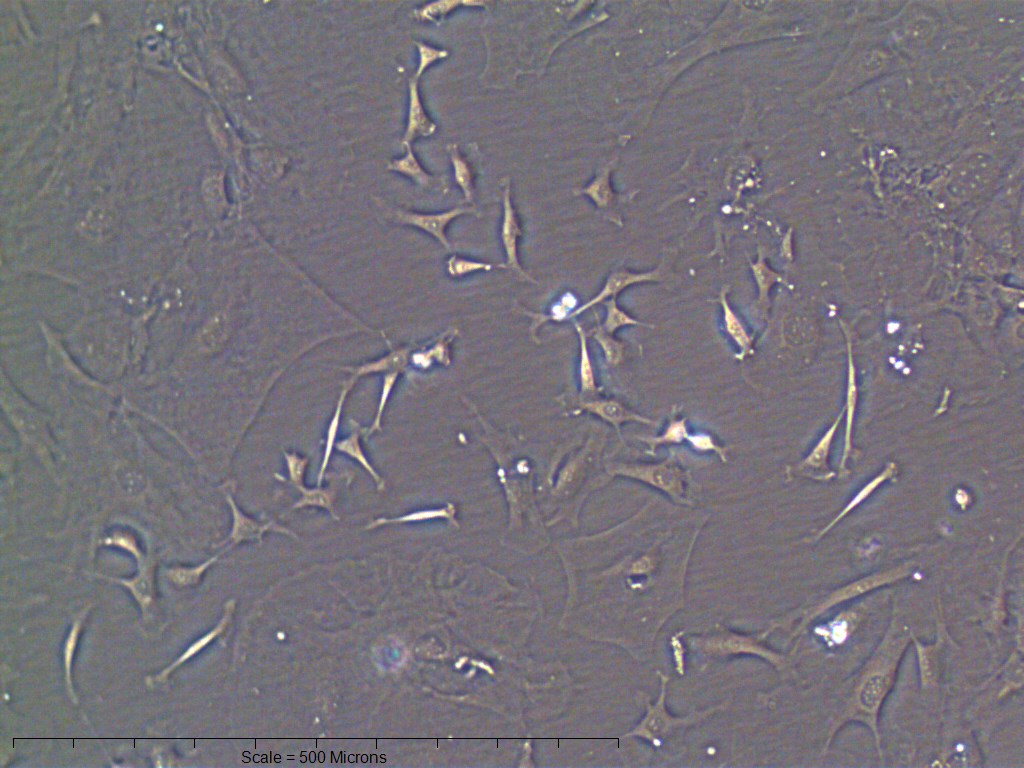

Supplement: Supplemental Information 4 [file peerj-06-5757-s004.jpg]

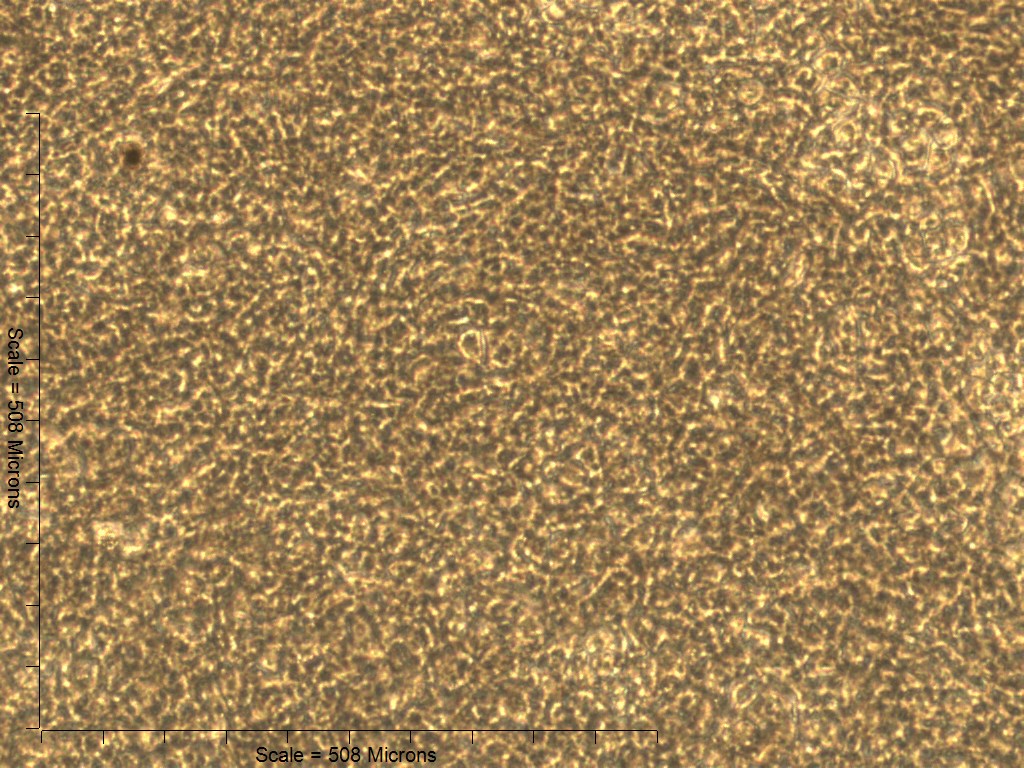

Supplement: Supplemental Information 6 [file peerj-06-5757-s006.jpg]

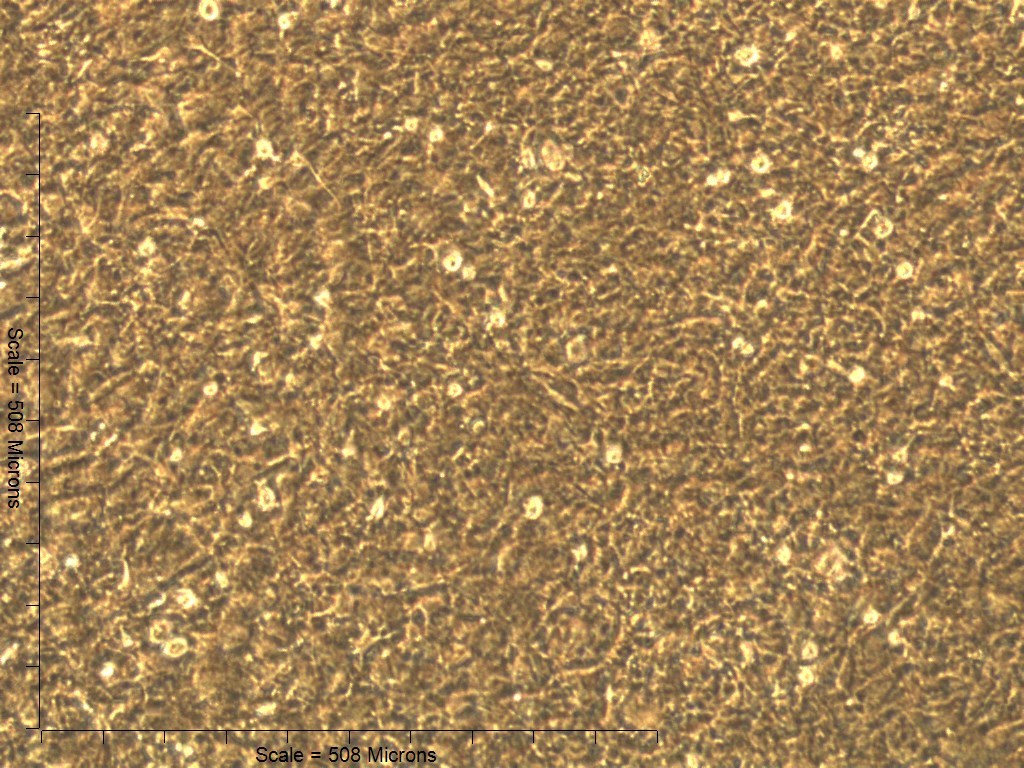

Supplement: Supplemental Information 7 [file peerj-06-5757-s007.jpg]

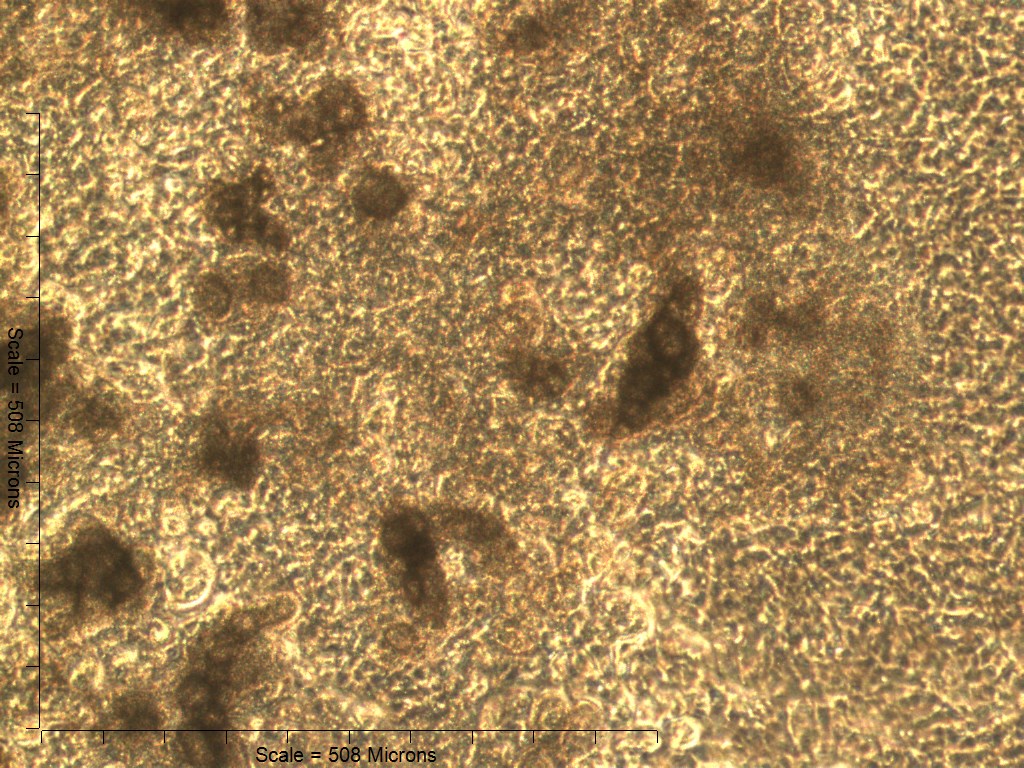

Supplement: Supplemental Information 8 [file peerj-06-5757-s008.jpg]

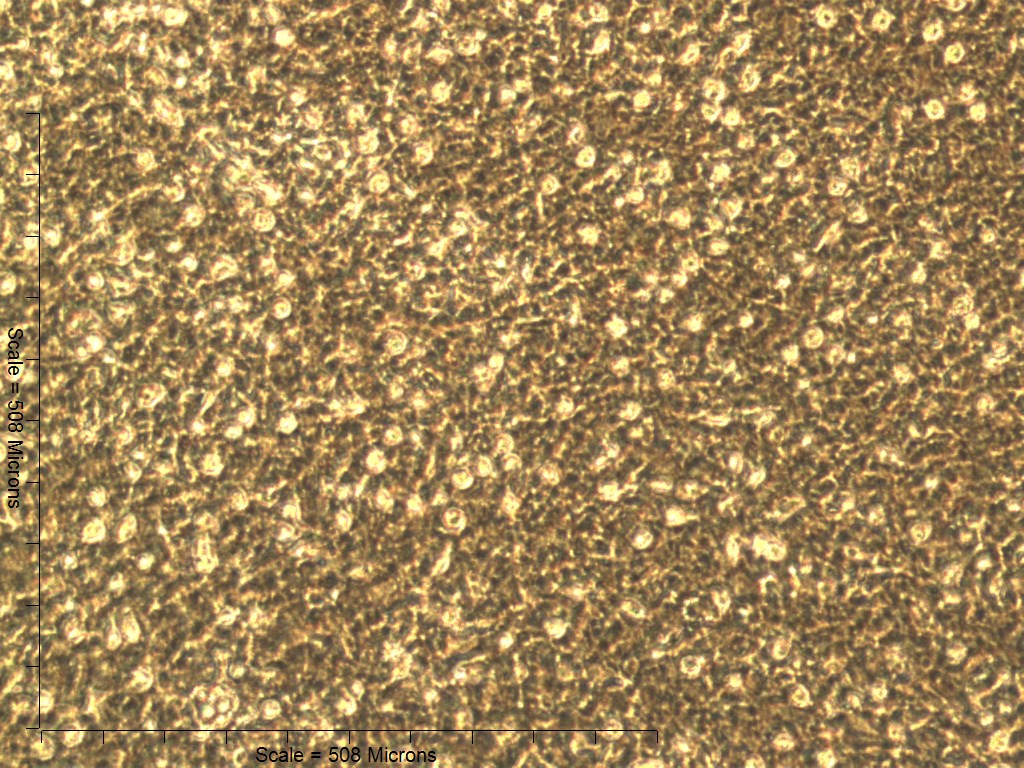

Supplement: Supplemental Information 9 [file peerj-06-5757-s009.jpg]

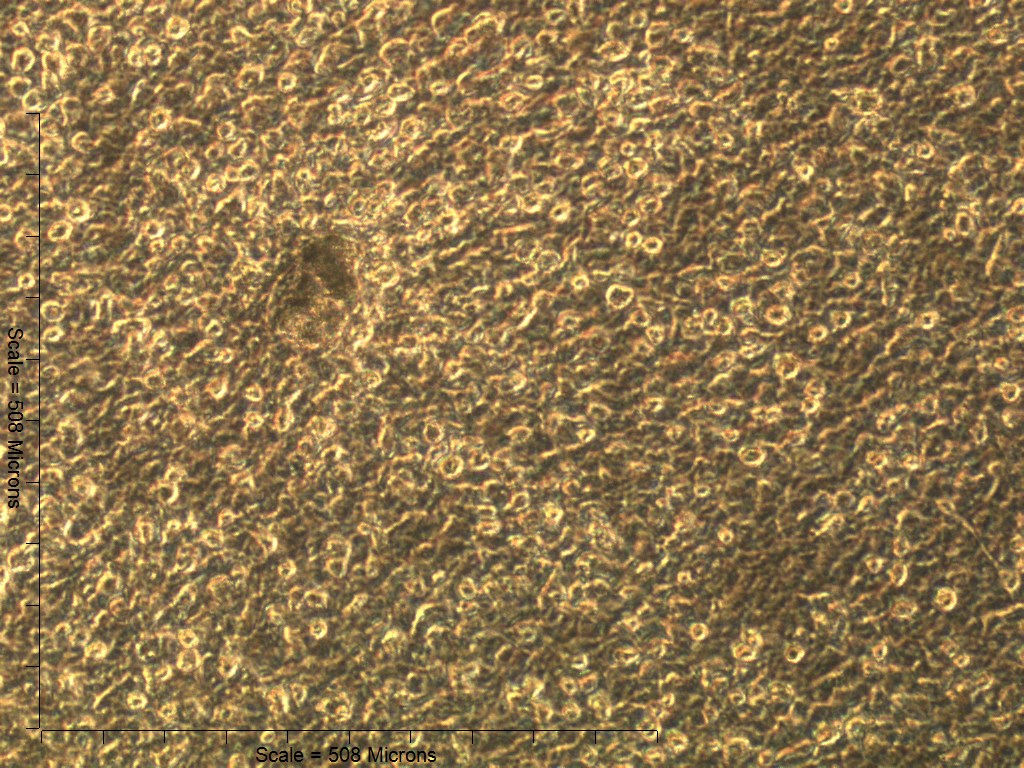

Supplement: Supplemental Information 10 [file peerj-06-5757-s010.jpg]

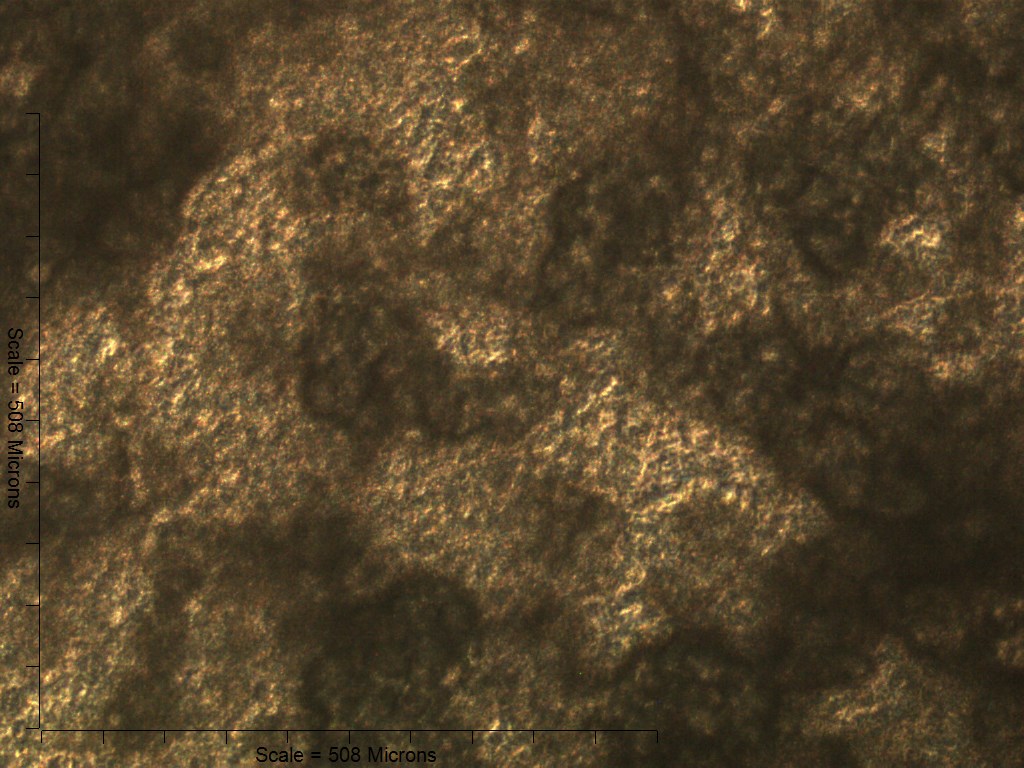

Supplement: Supplemental Information 11 [file peerj-06-5757-s011.jpg]

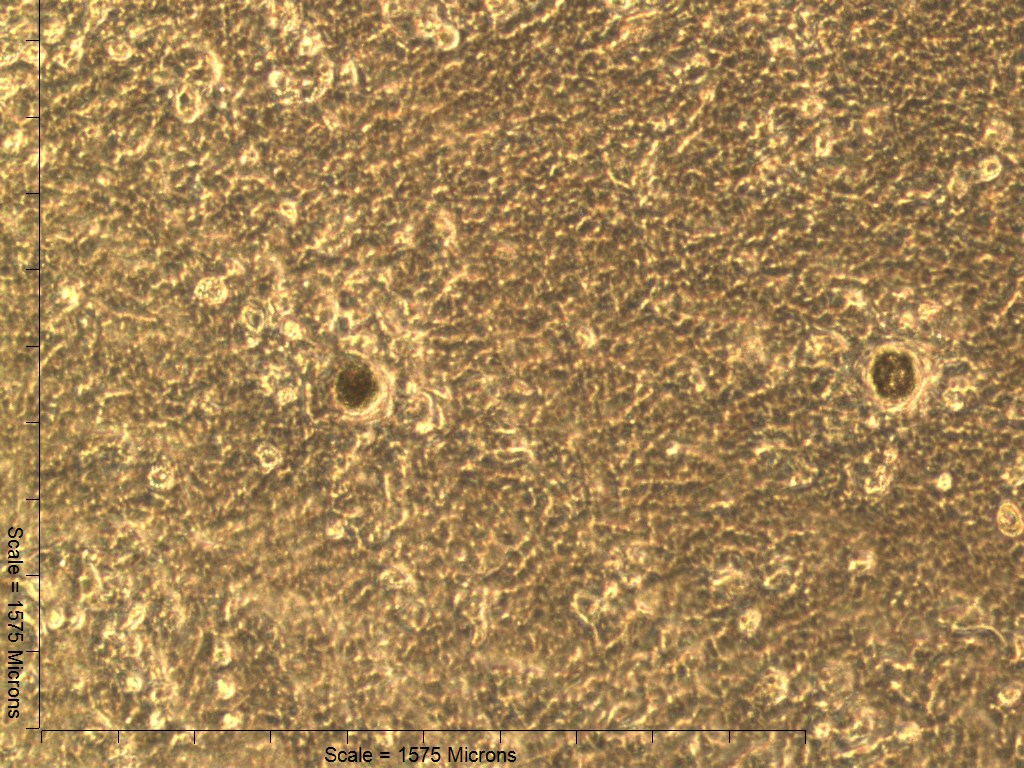

Supplement: Supplemental Information 12 [file peerj-06-5757-s012.jpg]

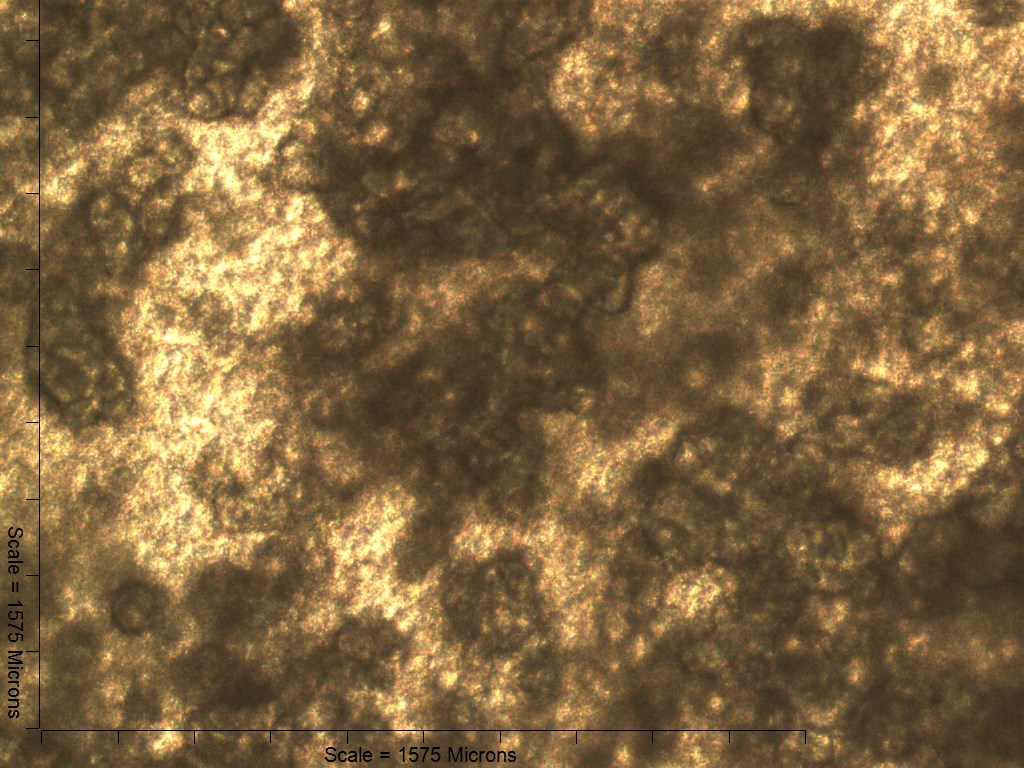

Supplement: Supplemental Information 13 [file peerj-06-5757-s013.jpg]

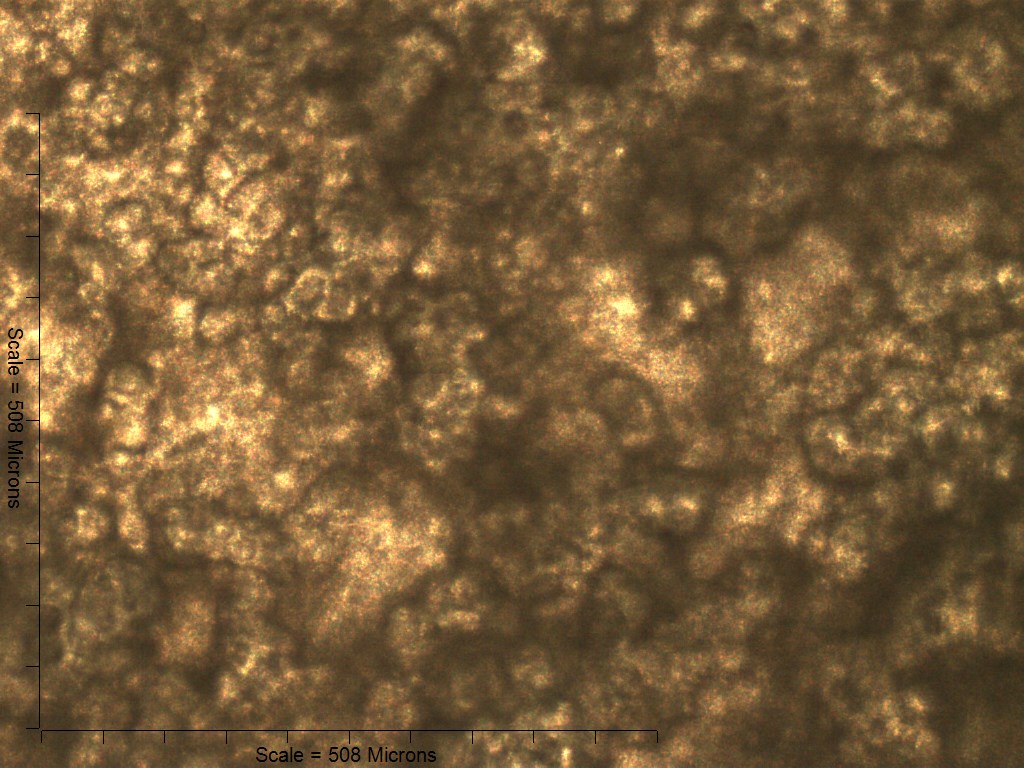

Supplement: Supplemental Information 14 [file peerj-06-5757-s014.jpg]

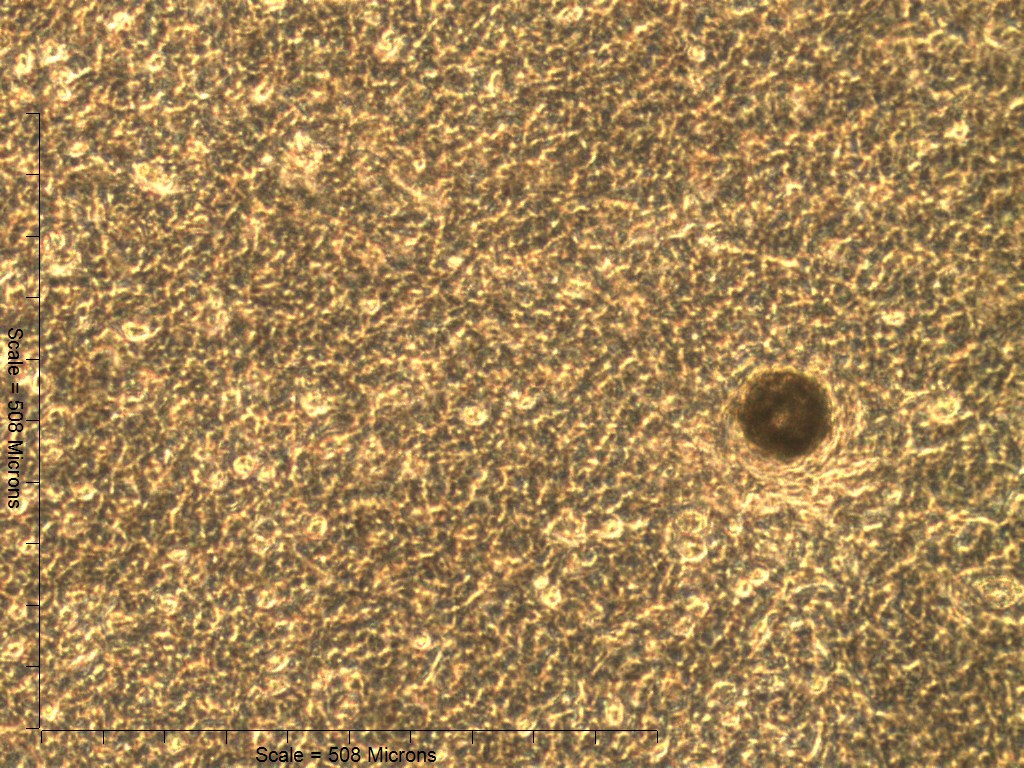

Supplement: Supplemental Information 15 [file peerj-06-5757-s015.jpg]

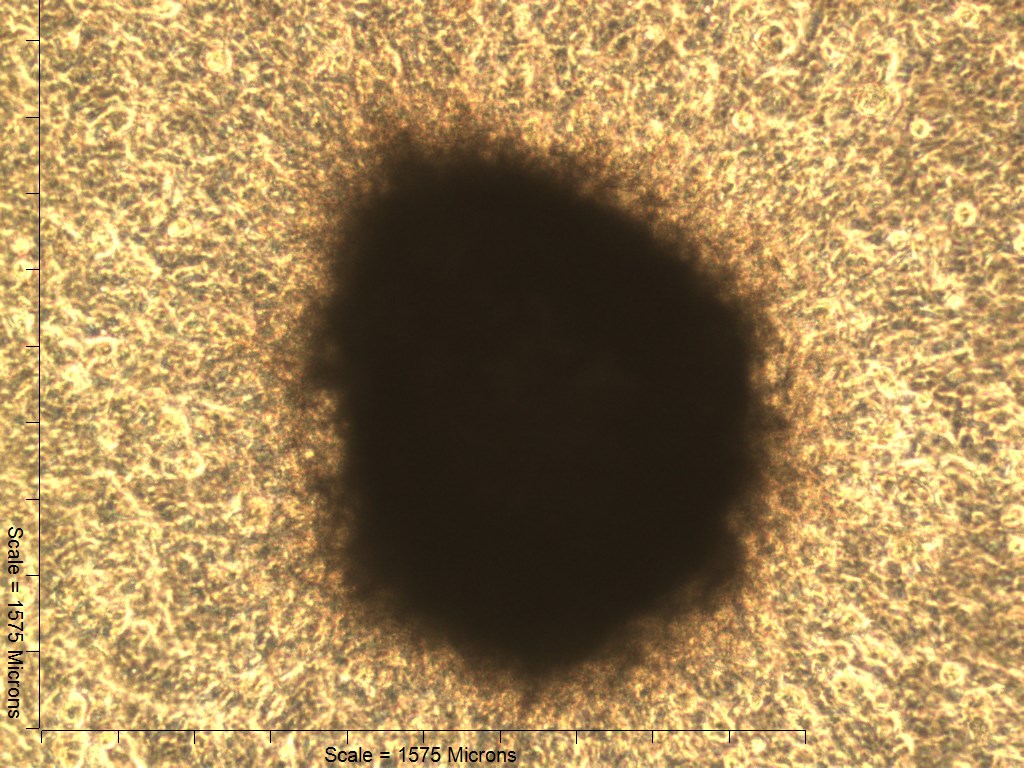

Supplement: Supplemental Information 16 [file peerj-06-5757-s016.jpg]

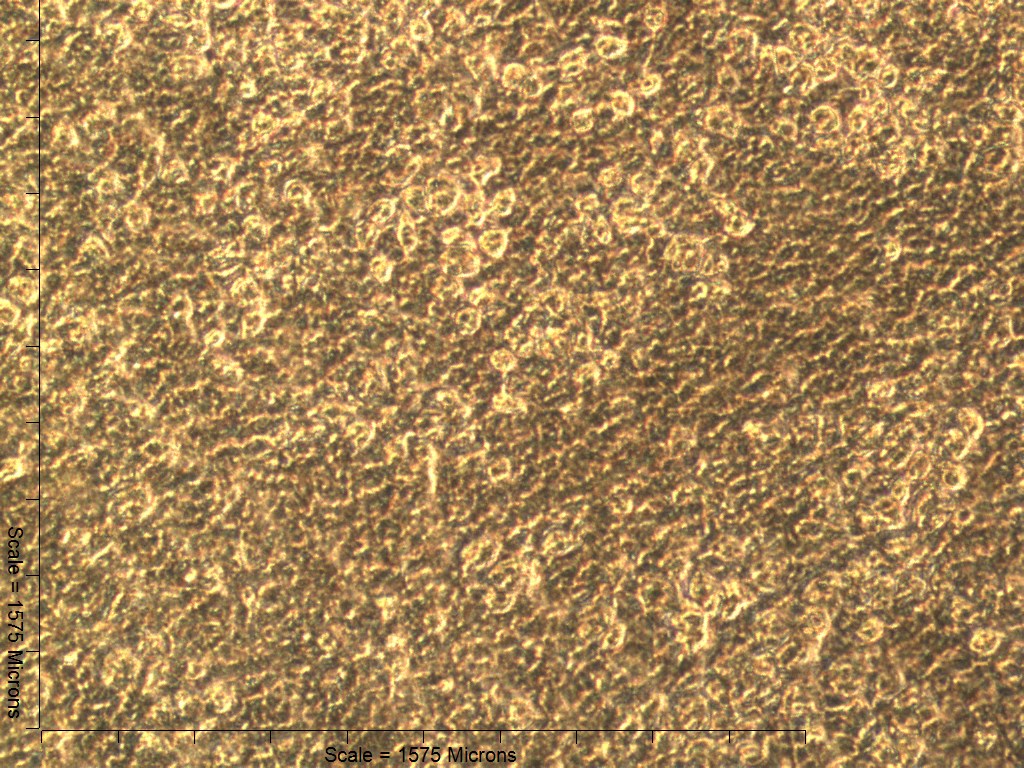

Supplement: Supplemental Information 17 [file peerj-06-5757-s017.jpg]

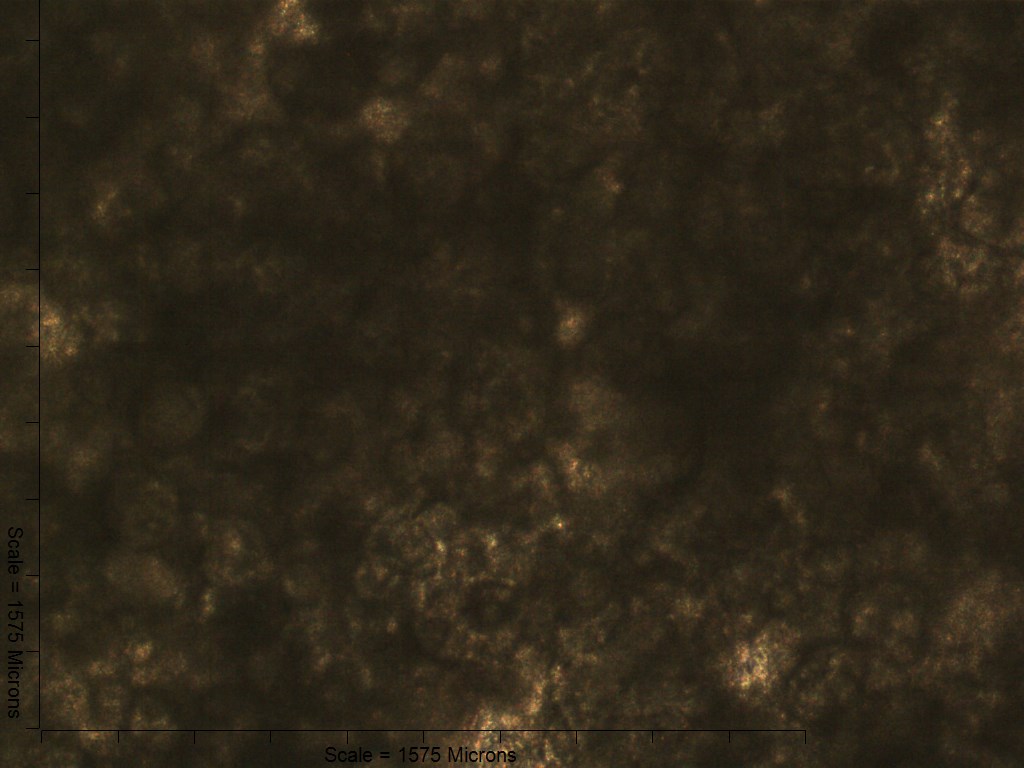

Supplement: Supplemental Information 18 [file peerj-06-5757-s018.jpg]

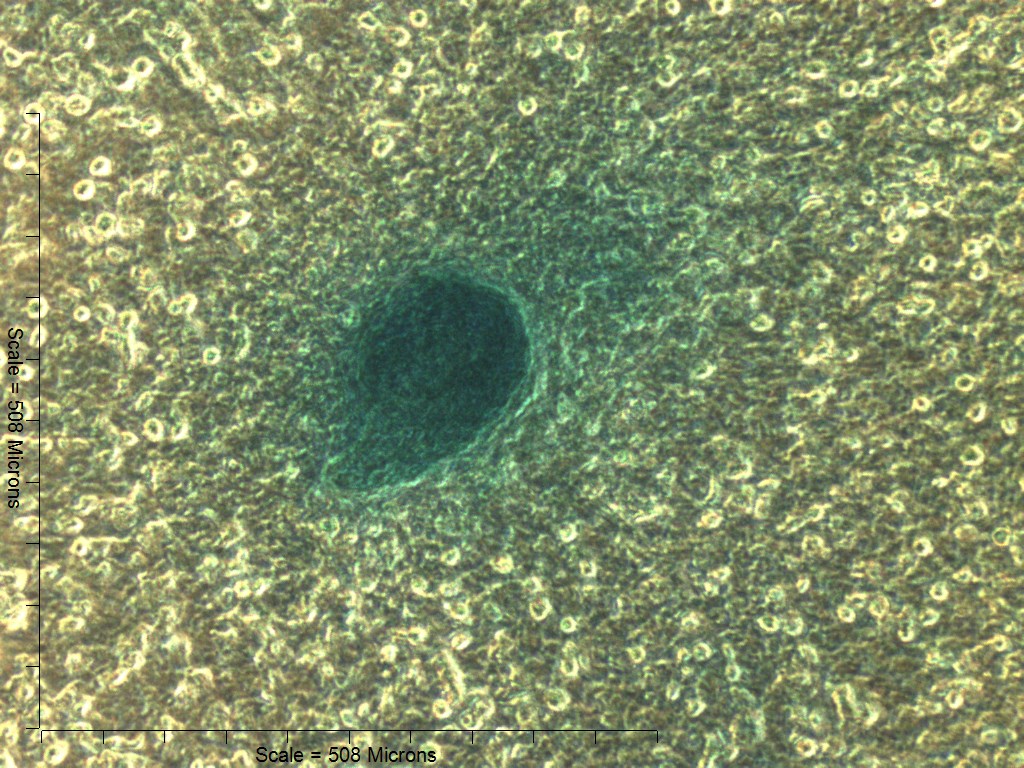

Supplement: Supplemental Information 19 [file peerj-06-5757-s019.jpg]

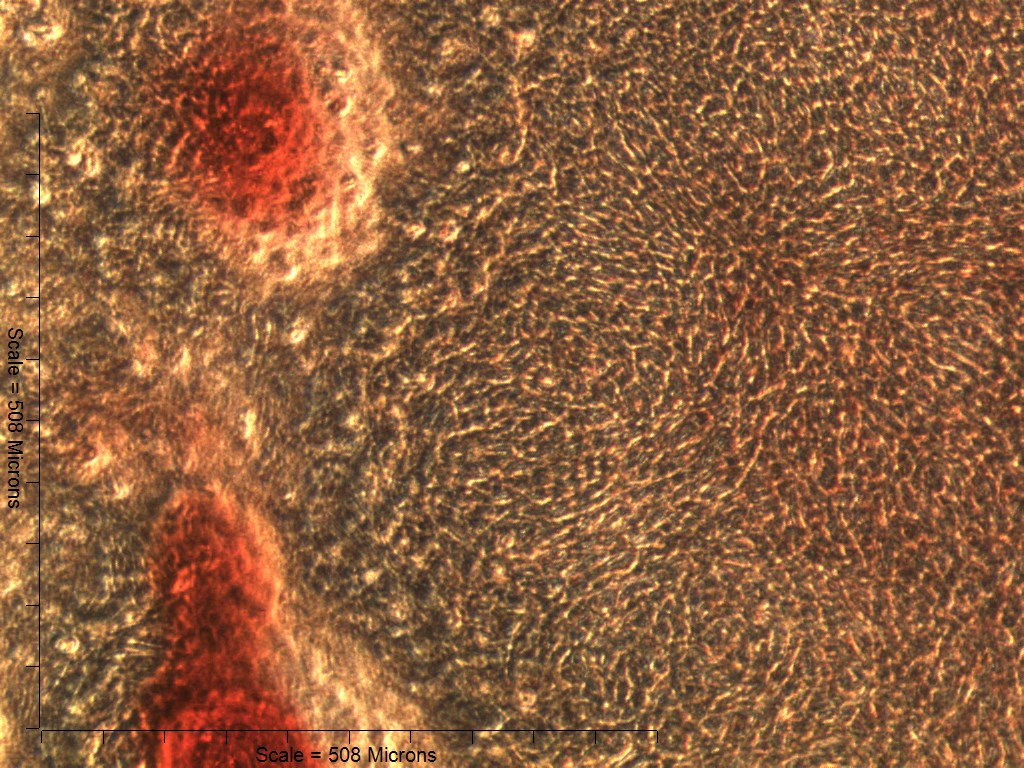

Supplement: Supplemental Information 20 [file peerj-06-5757-s020.jpg]

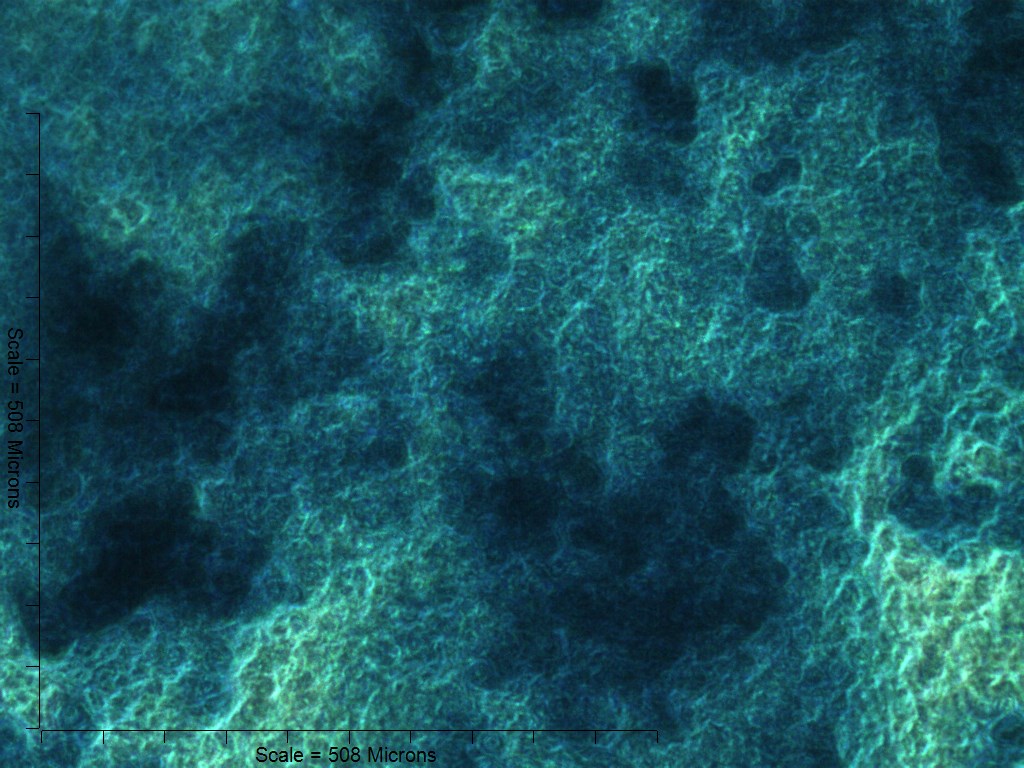

Supplement: Supplemental Information 21 [file peerj-06-5757-s021.jpg]

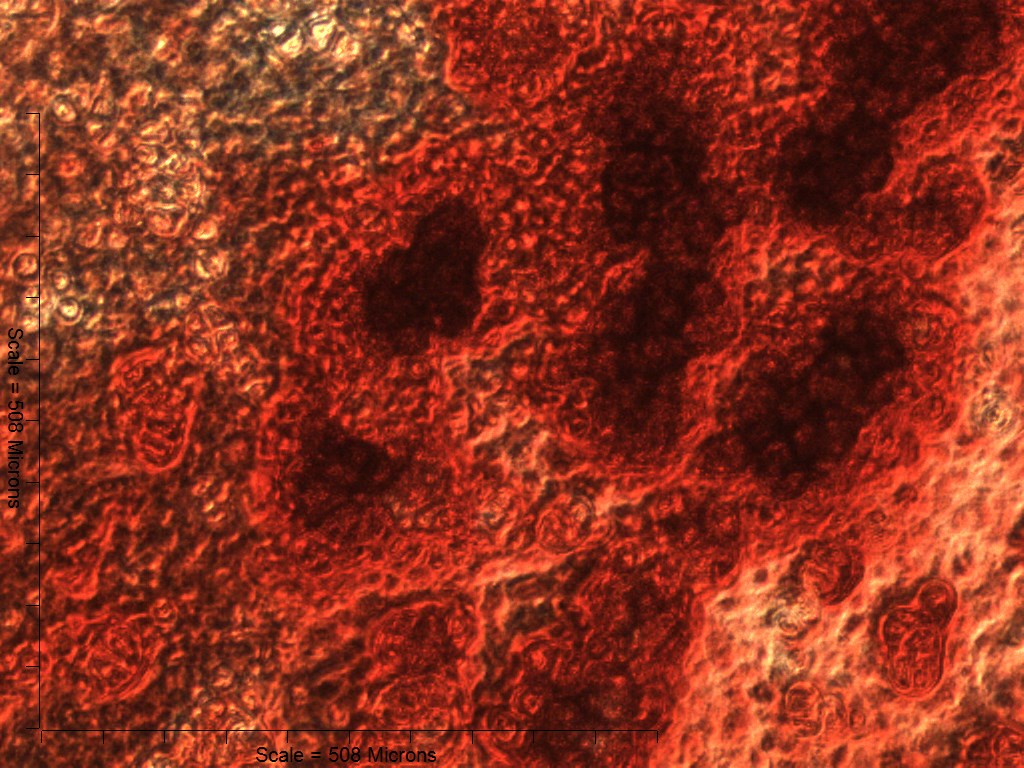

Supplement: Supplemental Information 22 [file peerj-06-5757-s022.jpg]

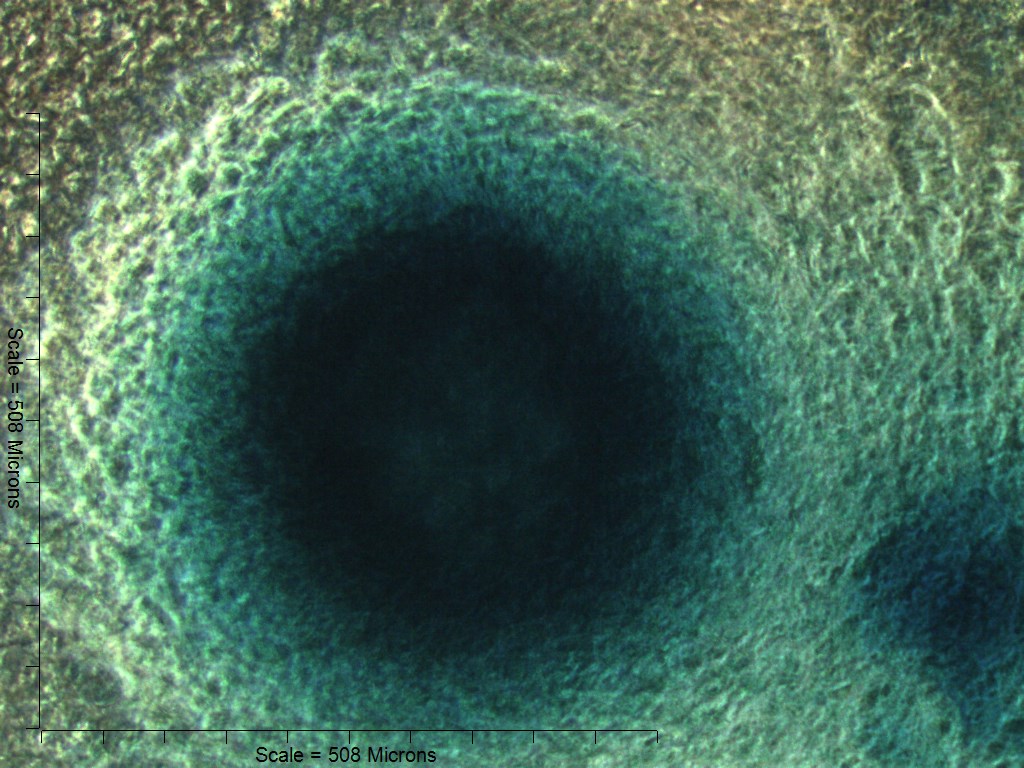

Supplement: Supplemental Information 23 [file peerj-06-5757-s023.jpg]

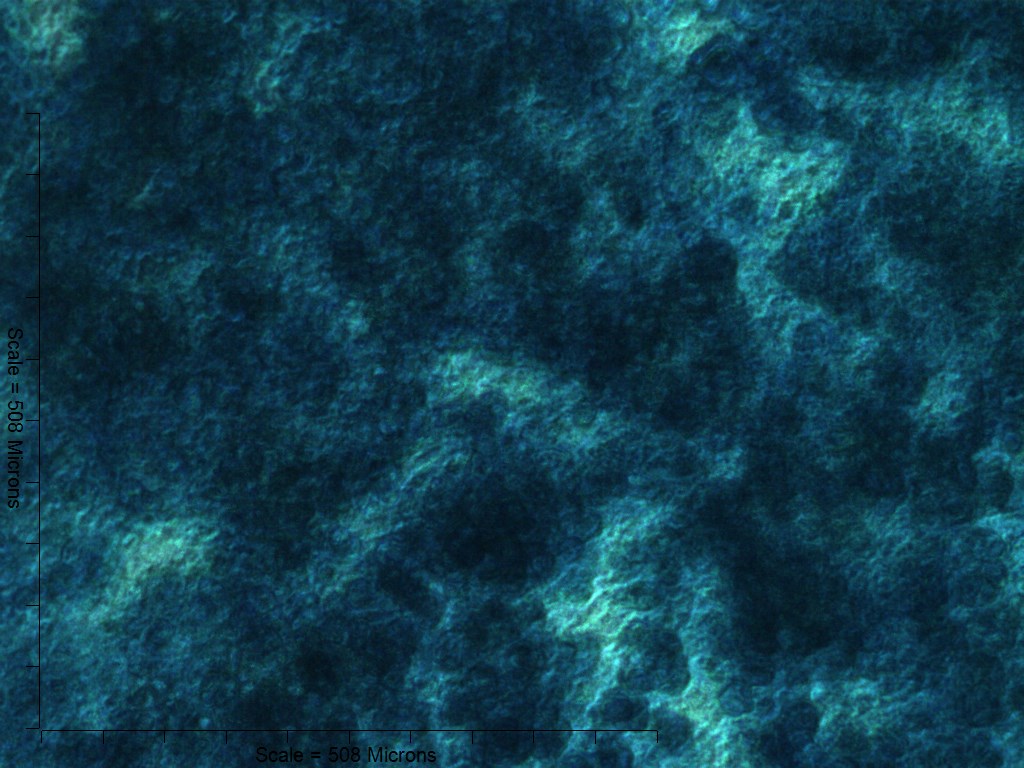

Supplement: Supplemental Information 24 [file peerj-06-5757-s024.jpg]

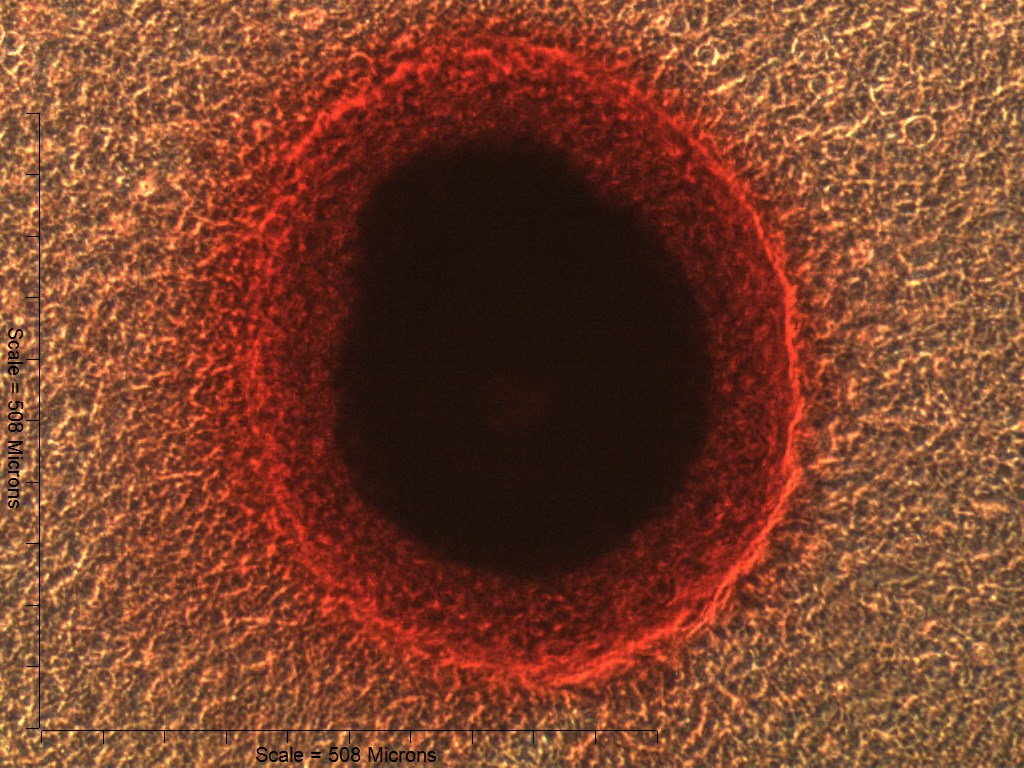

Supplement: Supplemental Information 25 [file peerj-06-5757-s025.jpg]

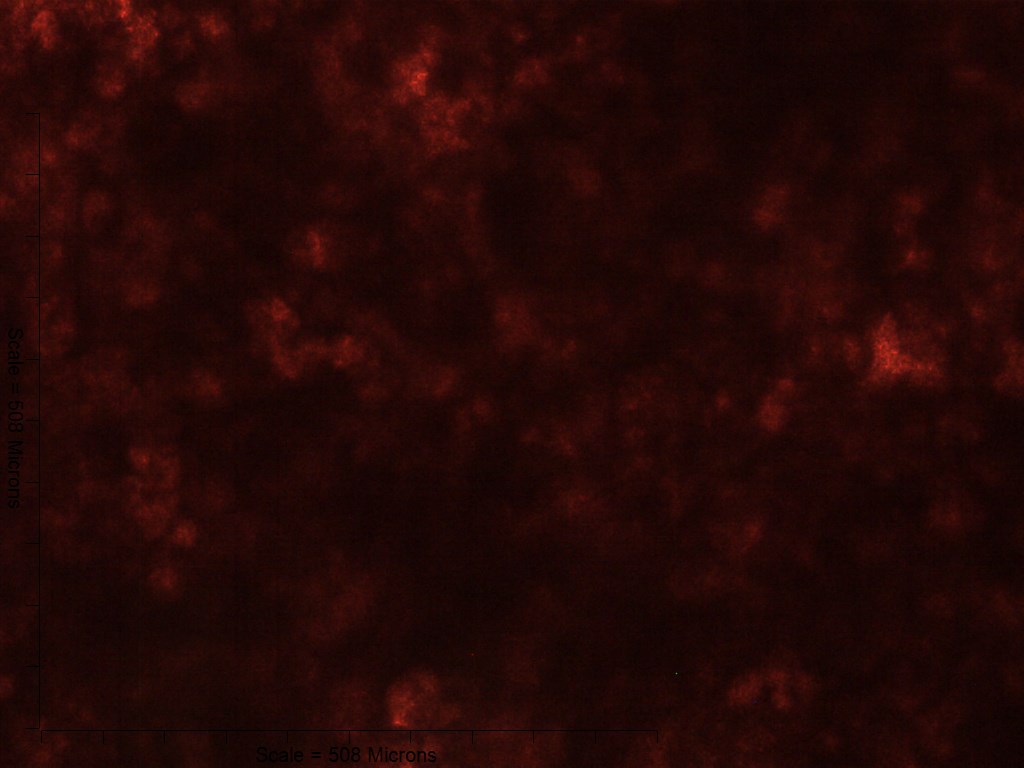

Supplement: Supplemental Information 26 [file peerj-06-5757-s026.jpg]
